# Deciphering the Transcription Factor-Dominated Ecosystem During Esophageal Squamous Cell Carcinoma Progression at the Single-Cell Level

**DOI:** 10.3390/ijms27104433

**Published:** 2026-05-15

**Authors:** Congxue Hu, Xinyu Li, Weixin Liang, Shujuan Li, Xiaozhi Huang, Jing Chen, Kaiyue Yang, Xia Li, Yunpeng Zhang, Jing Bai

**Affiliations:** 1 College of Bioinformatics Science and Technology, Harbin Medical University, Harbin 150081, China; hucx1996@hrbmu.edu.cn (C.H.); lixinyu@hrbmu.edu.cn (X.L.); liangwx_bio@163.com (W.L.); 2023020630@hrbmu.edu.cn (S.L.); huangxiaozhi1108@163.com (X.H.); chenjing_bio@163.com (J.C.); yangkaiyue0904@hrbmu.edu.cn (K.Y.); lixia@hrbmu.edu.cn (X.L.); 2 College of Biomedical Information and Engineering, Hainan Medical University, Haikou 570100, China

**Keywords:** esophageal squamous cell carcinoma, transcription factor regulation, single-cell RNA sequencing, tumor microenvironment, gene regulatory network

## Abstract

Esophageal squamous cell carcinoma (ESCC) progression involves dynamic cellular state transitions and tumor microenvironment remodeling, accompanied by extensive transcriptional regulation reprogramming. Here, we systematically mapped the TF-mediated regulatory landscape underlying ESCC progression at single-cell resolution by integrating stage-specific ESCC single-cell transcriptomic datasets comprising over 200,000 cells with TF–target interaction networks. Using a random walk algorithm combined with hypergeometric testing, we identified malignant progression-associated TFs (mpTFs) across multiple cell types and disease stages. Our analysis revealed extensive stage-dependent regulatory remodeling during ESCC progression. TCF4 was identified as an early-stage regulator associated with epithelial–mesenchymal transition activation and malignant invasive phenotypes. In immune lineages, BATF and IRF4 exhibited trajectory-associated activation during *CD4*^+^ T-cell differentiation and *CD8*^+^ T-cell exhaustion, suggesting critical roles in immunosuppressive T-cell state transitions. Additionally, mpTF-mediated remodeling of M2 macrophage subpopulations contributed to immunosuppressive tumor microenvironment formation during advanced ESCC progression. We further identified prognosis-associated cell-type-specific and shared mpTFs, including TFAP2C, which was associated with stabilized fibroblast and monocyte functional states and a less aggressive tumor microenvironment phenotype. Collectively, this study provides a comprehensive single-cell atlas of TF-mediated regulatory programs during ESCC progression and offers potential therapeutic targets for precision oncology.

## 1. Introduction

Esophageal cancer, particularly esophageal squamous cell carcinoma (ESCC), is a highly invasive and heterogeneous malignancy, with high morbidity and mortality, especially in China and East Asia [1,2]. Due to the lack of specific early symptoms, ESCC is often diagnosed at advanced stages, leading to a poor prognosis [3]. Precision staging of ESCC can assist in innovating and optimizing treatment strategies. However, comprehensive studies on its precise staging remain limited.

Single-cell RNA sequencing (scRNA-seq) is a breakthrough technology that can dissect cellular heterogeneity within tumors, reveal intercellular interactions in the tumor microenvironment (TME), and analyze the composition of the immune microenvironment within tumors [4,5]. Recent scRNA-seq studies have explored ESCC tumor cell heterogeneity [6,7] and immune suppression mechanisms within the TME [8]. Studies suggested that ESCC progression is linked to *ANXA1*/*FPR2* signaling mediated by epithelial cells and fibroblasts through receptor–ligand interactions [9] and myofibroblasts specifically enriched with *STAT1* [10]. However, it remains unclear how the characteristics of different molecules at the cellular level change dynamically during ESCC initiation and progression, and whether these changes are influenced by transcription factors (TFs) regulating specific cell types.

TFs are key molecules that regulate gene expression and play crucial roles in cell differentiation, functional maintenance, and cancer progression [11]. The transcriptional state of a cell derives from the underlying gene regulatory network (GRN), including TFs and their target genes. Traditional research methods are limited in elucidating the regulatory roles of TFs across different cell types and states, while scRNA-seq enables the characterization of expression patterns and regulatory networks of TFs at the single-cell level, aiding in resolving cellular heterogeneity and molecular mechanisms.

Recent studies have analyzed the activity and regulatory mechanisms of TFs in various cancer cell types. For example, the TF SREBPs are significantly activated in prostate cancer, affecting metabolic alterations and driving cancer development [12]. In addition, TFs such as YAP and TAZ play key roles in fibroblast phenotypic transformation and activation [13]. However, the dynamic effects of these TFs on cellular as well as molecular mechanisms during ESCC initiation and progression at the single-cell level remain unexplored.

In this study, we aimed to systematically characterize the dynamic TF-mediated regulatory landscape underlying ESCC progression at single-cell resolution, identify malignant progression-associated TFs across diverse cell types and disease stages, and elucidate the transcriptional mechanisms driving tumor evolution and tumor microenvironment remodeling during ESCC progression. To this end, we analyzed scRNA-seq data from 204,831 cells across 67 ESCC samples, including stage I, II, and III tumor samples and 11 adjacent normal tissue samples, and constructed a GRN encompassing 794 TFs and 9760 genes. Our analysis revealed transcriptomic changes in epithelial cells, fibroblasts, and immune cells (T lymphocytes and myeloid cells) during ESCC initiation and progression, accompanied by significant shifts in specific subcluster frequencies. Here, we identified cell-type-related mpTFs and systematically analyzed their dynamic activity changes and functional roles throughout the disease course. These mpTFs drove gene expression programs (GEPs) specific to malignant epithelial cells at different stages, promoting cancer progression. They also regulated the transition and functions of fibroblast subtypes, contributing to tumorigenesis, invasion, and metastasis through the secretion of cytokines and matrix metalloproteinases. Importantly, these mpTFs influenced immune cell differentiation, leading to the activation and differentiation of *CD4*^+^ T cells and exhaustion of *CD8*^+^ T cells, as well as phenotypic shifts in macrophages within the TME, enhancing immunosuppressive capacity and thus accelerating tumor progression. In addition, we identified both cell-type-specific and shared mpTFs, revealing the complex regulatory relationships within the ESCC TME. We found a series of mpTFs significantly associated with patient survival, which could serve as potential biomarkers for ESCC prognosis. In conclusion, our study provided a comprehensive atlas focused on TF regulatory networks underlying ESCC initiation and progression at single-cell resolution. This work provides a comprehensive single-cell atlas of TF-mediated regulatory networks during ESCC progression, systematically identifying malignant progression-associated TFs and their stage-dependent regulatory programs across malignant epithelial, stromal, and immune cell populations. These findings deepen our understanding of the transcriptional mechanisms underlying tumor evolution and tumor microenvironment remodeling, while offering potential biomarkers and therapeutic targets for precision oncology in ESCC.

## 2. Results

### 2.1. Constructing a Single-Cell Atlas of TF Regulatory Networks in the TME

**Figure 1:**
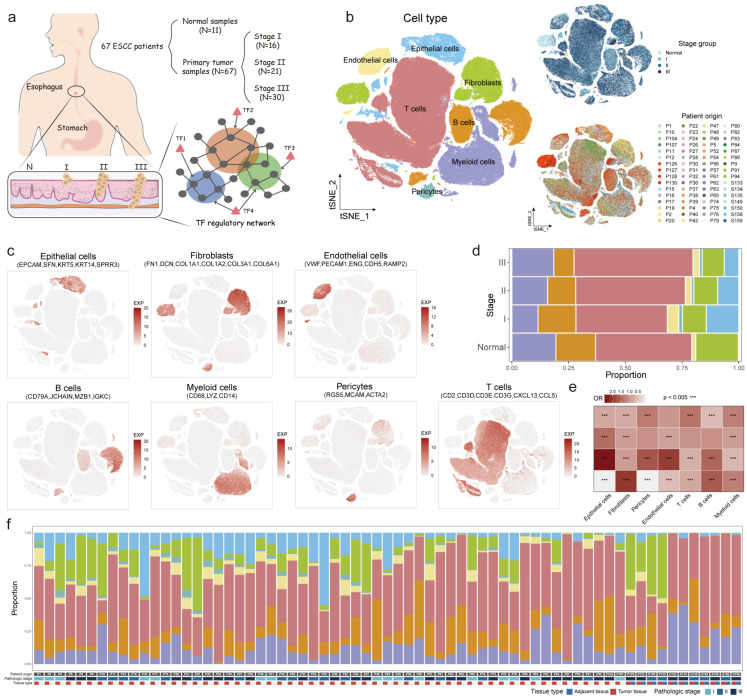
Single-cell atlas of 204,831 cells from Normal, stage I, II, and III ESCC samples. (**a**) Graphical overview of scRNA-seq data information from ESCC samples and TF regulatory network construction. (**b**) t-distributed stochastic neighbor embedding (tSNE) plots showing 204,831 cells of 67 ESCC patients across all four stages, colored by the major cell type, disease stage, and patient origin. (**c**) tSNE plots showing expression levels of the marker genes. (**d**) Bar plot showing proportions of seven major cell types in each stage (**left**), colored by cell types. (**e**) Heatmap showing the ORs of cell type occurring in each stage (**right**, *p* indicating enrichment significance level). (**f**) Bar plot showing proportions of seven major cell types in each patient, colored by cell types. The annotation of pathological stages and tissue types is shown at the bottom.

To characterize the transcriptome and GRNs during ESCC progression, we integrated scRNA-seq data from 204,831 cells across 67 ESCC samples, including stage I, II, and III tumor samples and 11 adjacent normal tissue samples (Figure 1a; Table S1). A comprehensive GRN, containing 794 TFs and 9760 genes, was constructed to explore the regulatory atlas. Seven major cell types were identified: epithelial cells (e.g., *EPCAM*, *SFN*, and *KRT5*), fibroblasts (e.g., *FN1*, *DCN*, and *COL1A1*), pericytes (e.g., *RGS5*, *MCAM*, and *ACTA2*), endothelial cells (e.g., *VWF*, *PECAM1*, and *ENG*), T cells (e.g., *CD2*, *CD3D*, and *CD3E*), B cells (e.g., *CD79A*, *JCHAIN*, and *MZB1*), and myeloid cells (e.g., *CD68*, *LYZ*, and *CD14*) (Figure 1b,c). T cells dominated all stages, while epithelial and B cell proportions decreased with progression. Conversely, myeloid cell proportions increased, and fibroblast proportions remained stable (Figure 1d,e). Stage I samples showed an enrichment of epithelial, pericyte, and endothelial cells, indicating early tissue remodeling [14]. Fibroblasts were most abundant in normal tissue samples, suggesting their role in maintaining tissue homeostasis [9]. Notably, substantial inter-patient variability highlights the heterogeneity in the TME (Figure 1f).

**Figure 2:**
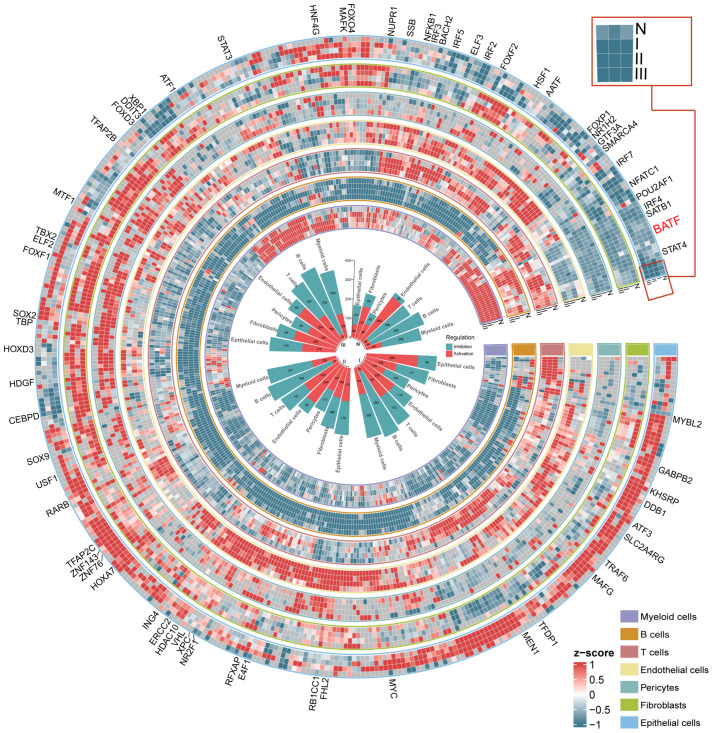
Transcription factor regulation atlas of 414 mpTFs across cell types and disease stages. The circular heatmap shows the activity levels of 414 mpTFs across four stages, with each circle representing a cell type (**outside**). The legend shows the color gradient of scaled activity expression (z-score > 0 representing activation, while z-score < 0 representing inhibition of TFs). The circular barplot shows the number of two mpTF regulations for seven cell types across four disease stages (**inside**).

Dynamic changes in TF activity were observed across different disease stages, significantly impacting the gene expression profiles of these cell types. The identified 414 mpTFs and their regulatory modules revealed distinct patterns across cell types and stages (Figure 2; Table S2; see Section 4). For example, TF activity in myeloid cells and T cells was predominantly downregulated in normal tissues, particularly in T cells, where activity was markedly inhibited (89.5%), reflecting their immunomodulatory roles. BATF, which was inhibited in most cell types during the normal stage, has previously been implicated in melanoma, where its expression in tumors promoted T-cell polarization toward Tregs, indicating a potential parallel function in ESCC [15].

In conclusion, we identified key mpTFs that regulated distinct cellular processes across various stages in the ESCC TME. These TFs were associated with tumor progression, immune evasion, and microenvironment remodeling, revealing complex transcriptional regulation mechanisms.

### 2.2. Dynamic Regulation Patterns of GEP-Related mpTFs During ESCC Progression

**Figure 3:**
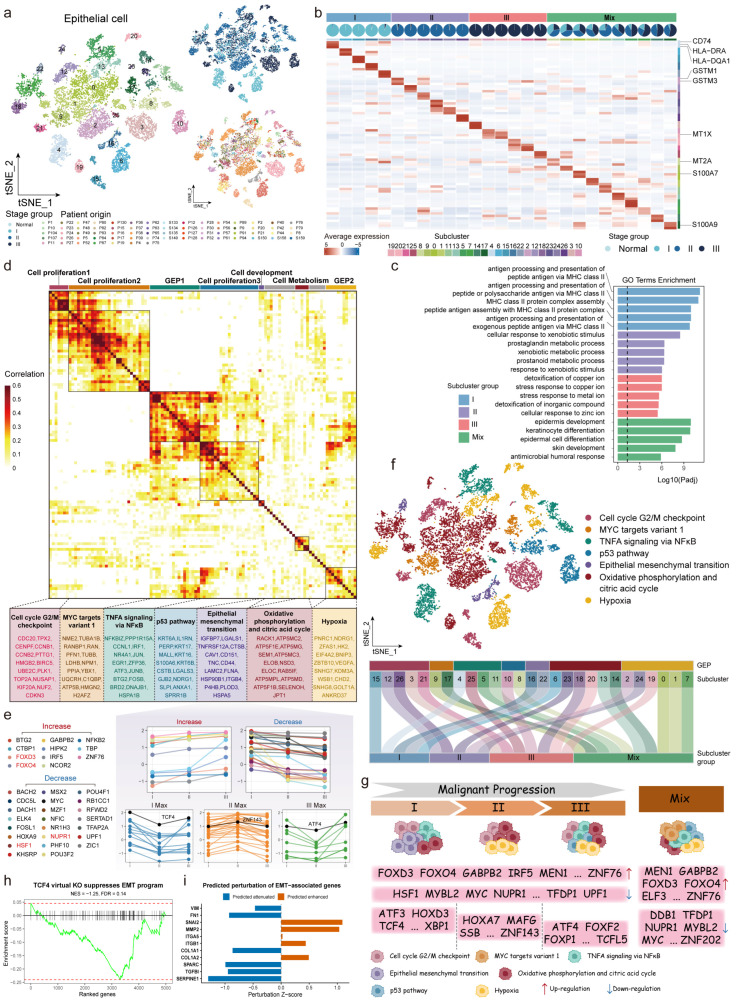
Characterization of malignant cell-related mpTFs in gene expression programs (GEPs). (**a**) tSNE plots showing 27 subclusters of malignant epithelial cells, colored by the subcluster, disease stage, and patient origin. (**b**) Heatmap showing the average expression of marker genes in each subcluster, ordered by subcluster groups. Pie charts showing the cancer stage composition of each subcluster. (**c**) Bar plot showing the top 5 GO terms enrichment in four subcluster groups. (**d**) Heatmap depicts pairwise correlations among 110 programs derived from 27 subclusters, identifying seven consistent GEPs in malignant epithelial cells, grouped by process category (**top**). Top 15 genes contributing to each GEP are listed (**bottom**). (**e**) Line plots showing the activity of mpTFs driving the EMT program across three cancer stages during ESCC progression. (**f**) tSNE plot showing 27 subclusters of malignant epithelial cells, colored by GEPs (**top**). Sankey diagram showing the distribution of seven GEPs in 27 subclusters and four subcluster groups, with bandwidths proportional to the allocation magnitude (**bottom**). (**g**) A graphical overview of mpTFs driving GEPs during cancer progression. MpTFs with the Increase and Decrease characteristics are indicated by up and down arrows, respectively. (**h**) Gene set enrichment analysis of the Hallmark EMT pathway following TCF4 virtual knockout. (**i**) Predicted perturbation of key EMT-associated genes following TCF4 virtual knockout.

ESCC is a major malignancy originating from the epithelial layer of the esophageal mucosa. To further investigate malignant epithelial cell heterogeneity, we reclustered epithelial cells and distinguished malignant cells from normal cells through copy number variation (CNV) (Figure S1a). A total of 14,413 malignant epithelial cells were identified and classified into 27 subclusters, which exhibited marked heterogeneity across disease stages (Figure 3a). Subclusters were categorized by the proportion of cells from each stage, with stage-specific clusters defined as those containing over 90% of cells from a single stage. Mixed clusters comprised cells from multiple stages. Five subclusters (4, 6, 15, 16, 22) were categorized as the stage I group, six subclusters (2, 12, 18, 23, 24, 26) were predominantly derived from stage II, and six subclusters (3, 10, 19, 20, 21, 25) were primarily sourced from stage III. The remaining subclusters (0, 1, 5, 7, 8, 9, 11, 13, 14, 17) formed a mixed group (Figure 3b). Transcriptome analysis revealed distinct expression patterns across subcluster groups. Malignant epithelial cells in stage I expressed *CD74*, *HLA-DRA*, and *HLA-DQA1*, significantly enriched in MHC class II antigen processing and presentation, which was associated with poor prognosis in ESCC [16]. Stage II cells showed specific expression of *GSTM1* and *GSTM3*, involved in external stimuli response and prostaglandin metabolism, with *GSTM1* being a known risk factor in renal carcinoma [17]. Stage III cells expressed *MT1X* and *MT2A*, linked to copper ion response and cancer prognosis [18]. The mixed group expressed *S100A7* and *S100A9*, influencing epithelial differentiation, migration, and invasion across cancers (Figure 3b,c).

To explore functional heterogeneity, we applied non-negative matrix factorization (NMF) to the malignant epithelial cell gene expression matrix, identifying seven gene expression programs (GEPs) representing specific functions and cellular states (Section 4, Figure 3d, Table S3). These GEPs corresponded to biological processes, including proliferation, metabolism, epithelial–mesenchymal transition (EMT), TNFA signaling via NFκB, and hypoxia. The G2M checkpoint program was enriched in proliferation markers such as *CDC20* and *CCNB1*, while the MYC target V1 program, also linked to proliferation, expressed *RANBP1*, correlating with poor prognosis [19]. The EMT program, a hallmark of cancer progression, was characterized by *LGALS1* and *IGFBP7*, which act as key regulators of invasion and differentiation. The hypoxia program, enriched in *VEGFA*, promoted angiogenesis and immune evasion [20]. Having identified key GEPs driving functional heterogeneity, we further explored the TFs regulating these programs across ESCC stages. Based on dynamic changes in activity scores, 270 malignant epithelial-related MT-TFs were classified into five categories: (i) TFs with activity scores gradually increasing (Increase), (ii) TFs with activity scores gradually decreasing (Decrease), and (iii) TFs with peak activity scores specifically observed in stage I, II, or III, respectively (Figure 3e, Table S4). FOXD3 and FOXO4 exhibited increasing activity across stages, regulating proliferation and EMT suppression. In contrast, TFs like HSF1 and NUPR1 showed decreasing activity, among which HSF1 enhanced cancer cell survival and resistance to programmed cell death [21]. TFs with stage-specific peak activities, such as TCF4 (I max), ZNF143 (II max), and ATF4 (III max), drove critical processes like EMT initiation, cell cycle regulation, and redox balance [22]. Notably, TCF4 was associated with EMT initiation in early stages, potentially enhancing invasion capacity [23]. In advanced ESCC, ATF4 regulated *VEGFA* expression, which promoted angiogenesis and hypoxia adaptation by enhancing endothelial cell survival and facilitating immune evasion.

In addition, we analyzed the specific enrichment of seven GEPs across subcluster groups (Figure 3f). The MYC target V1 program, enriched in subclusters 9 and 17 of the mixed group, demonstrated broad regulatory potential across disease stages. MYBL2, an oncogenic TF with the Decrease characteristic, was primarily involved in regulating the cell cycle and proliferation within the mixed group [24]. NUPR1, another TF also with the Decrease characteristic, was linked to proliferation and metastasis in diverse cancers [25,26]. MEN1, a TF with Increase characteristic, was identified as an oncogene driving cancer progression. The EMT program, enriched in subclusters 16 and 22 of stage I, highlighted the critical role of TFs in early-stage tumor invasion. TCF4, an I max signature TF, promoted cell proliferation and EMT initiation by activating target gene transcription [23]. Oxidative phosphorylation, enriched across all stages, underscored the importance of TF-driven metabolism in ESCC progression (Figure 3g). To further investigate the potential regulatory role of TCF4 in EMT-associated malignant phenotypes, we performed virtual knockout experiments in early-stage malignant epithelial cells. Virtual knockout of *TCF4* induced widespread and stable transcriptional perturbations (Figure S1b,c) [27]. We further revealed a negative enrichment trend of the Hallmark EMT pathway following *TCF4* virtual knockout (NES = −1.25, FDR = 0.14), suggesting a potential attenuation of EMT-associated transcriptional programs (Figure 3h). At the gene level, multiple canonical EMT and invasion-related markers, including *VIM*, *FN1*, *SNAI2*, *ITGA5*, and *MMP2*, exhibited consistent negative perturbation Z-scores, indicating predicted suppression upon *TCF4* depletion (Figure 3i). Furthermore, gene module analysis demonstrated coordinated negative perturbation across EMT, migration, and extracellular matrix remodeling gene sets, supporting a potential role of TCF4 in maintaining these malignant transcriptional programs (Figure S1d). Consistently, analysis of an independent early-stage ESCC bulk transcriptomic dataset (GSE213565 [28]) revealed that TCF4 activity scores were positively correlated with EMT, invasion, and malignancy-related signatures (Figure S1e), further supporting the association between TCF4 and aggressive phenotypic programs. These results provide additional evidence supporting a plausible role of TCF4 in regulating EMT-associated programs during early ESCC progression.

In summary, we systematically analyzed the TFs driving GEPs in malignant epithelial cells, revealing their roles in proliferation, invasion, and metabolic reprogramming across disease stages. These insights provide a foundation for precision therapies targeting stage-specific TFs.

### 2.3. Stage Preferences of Fibroblast Subtypes During ESCC Progression

**Figure 4:**
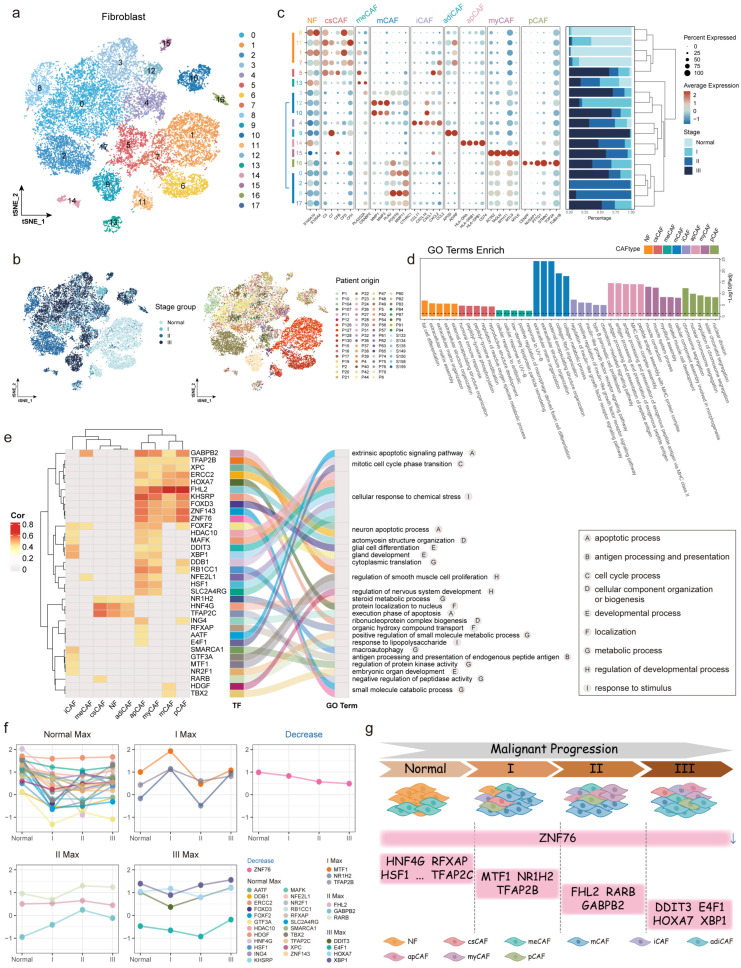
Heterogeneity of fibroblast subtypes in ESCC progression. (**a**,**b**) tSNE plots showing 18 subclusters of fibroblasts, colored by the subcluster (**a**), disease stage and patient origin (**b**). (**c**) Bubble plot of marker gene expression in fibroblasts. Bubble size represents the percent of cells expressing the gene and color indicates scaled average expression. Bar plot shows the proportion of four stages in each subcluster. (**d**) Bar plot of top 5 GO terms enrichment in nine fibroblast subtypes. (**e**) Clustered heatmap showing correlations between 34 mpTFs and fibroblast subtypes (**left**), with sankey diagram showing GO terms enriched by 34 mpTFs (**right**). The top GO term for each mpTF is shown, with categories indicated by letters. (**f**) Line plots showing mpTF activity across four disease stages, grouped into five categories and colored by mpTFs. (**g**) A graphical overview of mpTFs regulating fibroblast subtypes with stage preferences. MpTFs with the decrease characteristic are indicated by the down arrow.

Fibroblasts in ESCC progression promoted tumor growth, invasion, immune evasion, and contributed to extracellular matrix (ECM) remodeling, angiogenesis, and immune microenvironment regulation [29]. Analysis of 23,298 fibroblasts identified 18 subclusters (Figure 4a,b), which were further grouped into nine fibroblast subtypes associated with disease progression (Figure 4c). Normal fibroblasts (NF), characterized by *S100A4* and *S100A10* expression, maintained tissue homeostasis but could drive tumor invasion when dysregulated [30]. The role of *S100A4* in ECM assembly suggested early involvement in tumor microenvironment remodeling (Figure 4d). Complement-secreting CAFs (csCAF) supported humoral immunity by expressing complement factors like *C3*, *C7*, and *CFD* [31]. Metabolic CAFs (meCAF), expressing *PLA2G2A* and *CRABP2*, responded to cellular stimuli, and matrix CAFs (mCAF) exhibited stage-specific origins. mCAF with high *MMP1*, *MMP3*, and *PLAU* expression predominantly originates from stages I and III, promoting apoptosis, angiogenesis, and invasive growth. In contrast, *POSTN*- and *CTHRC*-expressing mCAF originating from stage II-mediated fibrosis and ECM remodeling [32]. Inflammatory CAFs (iCAF) promoted an immunosuppressive microenvironment via cytokines like *CCL2* and *CXCL14*, activating pathways such as STAT3/CCL2. Adipogenic CAFs (adiCAF), marked by *APOD* and *ADIRF*, predominantly originated from stage III tumors, while antigen-presenting CAFs (apCAF) expressed MHC-II molecules, supporting antigen processing, presentation, and immune regulation. Myofibroblasts (myCAF) exhibited high expression of *MYL9* and *MYLK*, facilitated ECM remodeling and invasion [33]. Proliferative CAFs (pCAF) expressed genes like *CENPF* and *NUSAP1*, promoting tumor proliferation. These findings highlighted fibroblast heterogeneity and diverse roles in tumor progression.

To characterize the regulatory mechanisms of mpTFs in fibroblast subtypes, we analyzed the correlation between 135 fibroblast-related mpTFs and nine fibroblast subtypes, ultimately identifying 34 key TFs that were significantly correlated with fibroblast subtypes (cor > 0.4, *p* < 0.05, Figure 4e). Similar regulatory patterns were observed in apCAF and myCAF. Shared TFs, such as HDAC10, MAFK, and HSF1, regulated apCAF and myCAF functions by modulating responses to inflammatory stimuli and stress tolerance mechanisms. Similarly, mCAF and pCAF exhibited a shared regulatory pattern, with TFs like GABPB2, TFAP2B, XPC, and ERCC2 linked to biological processes such as cell development, apoptosis, metabolism, and cellular component organization or biogenesis. Notably, RFXAP, a subtype-specific TF, was active in apCAF and participated in antigen processing and presentation. NR1H2, HNF4G, and TFAP2C were specifically identified in NF, csCAF, and adiCAF, suggesting their roles in precancerous or advanced stages of ESCC. Furthermore, we classified these 34 TFs into five categories based on changes in their activity across disease stages, including TFs with gradually decreasing activity scores (Decrease) as well as those peaking in Normal, I, II, or III stages (Figure 4f). Most TFs exhibited the highest activity in the normal stage, suggesting their roles in regulating the activation of normal fibroblasts into CAFs in adjacent normal tissues. For instance, HSF1 activated fibroblasts by targeting DKK3 expression [34]. Interestingly, ZNF76 was the only TF exhibiting decreasing activity and regulated apCAF, myCAF, mCAF, and pCAF. Its reduced expression correlated with platinum resistance [35], suggesting that ZNF76 might modulate chemotherapy sensitivity and could serve as a novel therapeutic target in ESCC. Further analysis revealed that TFs within specific subtypes closely correlated with disease stages. For example, FHL2, identified as an II Max signature TF, was significantly associated with mCAF. Since some mCAF subclusters originated from stage II samples, FHL2 likely promoted tumor progression in the middle stages by regulating the immunosuppressive functions of mCAF. Studies have shown that high expression of FHL2 in CAFs accelerated lung adenocarcinoma progression by promoting angiogenesis [36]. Additionally, the III Max signature TFs DDIT3 and XBP1 were highly correlated with iCAF, suggesting that these TFs influenced tumor progression by regulating immune responses and inflammatory signaling in iCAF (Figure 4g).

In conclusion, we identified nine functionally specific fibroblast subtypes and their mpTF regulatory networks during ESCC progression. This revealed the multidimensional regulatory roles of these TFs within the TME, providing a foundation for future therapeutic interventions targeting fibroblast-associated subtypes.

### 2.4. Mediating T Lymphocyte Fate Determination Through mpTFs

**Figure 5:**
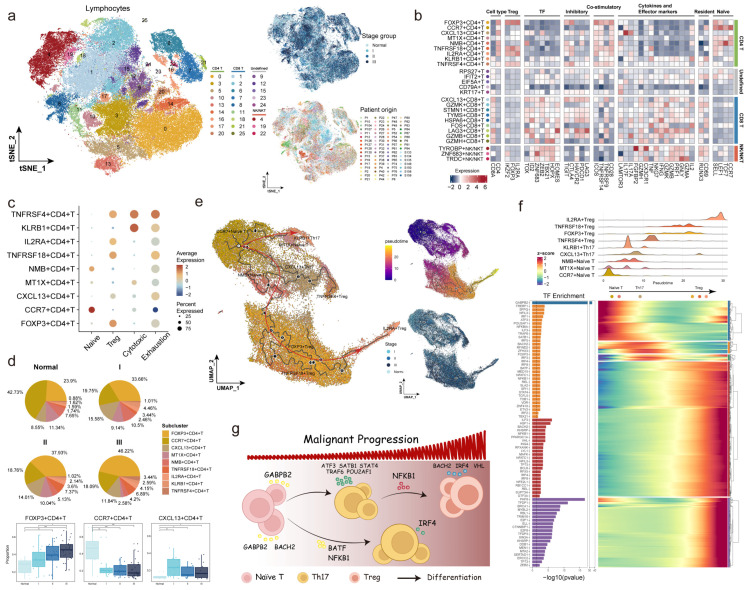
T cell-related mpTFs in altered CD4^+^ T cell fate. (**a**) tSNE plots showing 26 subclusters of T lymphocytes, colored by the subcluster, disease stage, and patient origin. (**b**) Heatmap showing the expression of signature genes associated with T cell function in each subcluster. (**c**) Bubble plot showing average expression of various signatures in *CD4*^+^ T cell populations. (**d**) Pie charts showing proportions of *CD4*^+^ T subclusters in each disease stage (**top**), and box plots showing proportions of *FOXP3*+, *CCR7*+, and *CXCL13*+*CD4*^+^ T in samples from different stages (**bottom**). Statistical significance was calculated by paired *t*-test. (**e**) Uniform Manifold Approximation and Projection (UMAP) plots showing the pseudotemporal trajectory of CD4^+^ T subclusters, predicted by R package monocle3, with red arrows showing trajectory direction, colored by subclusters (**left**) and stages (**bottom right**). Pseudotime coloring from purple to yellow (**top right**). (**f**) Heatmap showing the dynamic gene expression changes associated with *CD4*^+^ T subclusters in naïve T to Treg cell trajectory, with the density plot of the distribution of *CD4*^+^ T subclusters along the pseudotime (**top**). Bar plot showing the enrichment of T cell-related mpTFs in genes associated with the *CD4*^+^ T cell trajectory. (**g**) A graphical overview of mpTFs mediating *CD4*^+^ T cell fate transition.

Lymphocytes represented critical components of the TME in ESCC, playing pivotal roles in tumor immune surveillance and evasion. We further reclustered 98,474 lymphocytes, identifying 26 subclusters comprising nine *CD4*^+^ T cell populations (characterized by *CD4*, *FOXP3*, and *CCR7*), nine *CD8*^+^ T cell populations (characterized by *CD8A*, *CXCL13*, and *GZMK*), and three NK/NKT cell populations (characterized by *TYROBP*, *ZNF683*, and *TRDC*). We analyzed the expression patterns of functional marker genes to profile each cluster’s functional states and potential immune regulatory roles (Figure 5a,b). *CCR7*^+^, *MT1X*^+^, and *NMB*^+^ *CD4*^+^ T cells exhibited naïve T cell characteristics, with high expression of naïve signature and low expression of pro-inflammatory cytokines and effector molecules. These cells were predominantly in adjacent normal tissues and decreased proportionally with tumor progression (Figure 5c,d). Regulatory CD4^+^ T cells (Tregs, including *FOXP3*^+^, *TNFRSF18*^+^, *IL2RA*^+^, and *TNFRSF4*^+^ subpopulations) exhibited high levels of classic regulatory genes (e.g., *FOXP3* and *IL2RA*) and co-stimulatory molecules (e.g., *ICOS* and *CD28*), indicating strong immunosuppressive and regulatory functions. The proportion of Tregs increased significantly in tumor tissues, contributing to an immunosuppressive TME. *CXCL13*^+^ and *KLRB1*^+^ *CD4*^+^ T cells, representative of Th17 cells, expressed pro-inflammatory cytokines IL17A and IL17F, contributing to immune regulation and anti-tumor responses. These Th17 cells potentially activated other immune cells, such as neutrophils and macrophages, promoting anti-tumor immunity in early ESCC stages.

Pseudotime trajectory analysis identified two primary differentiation pathways for *CD4*^+^ T cells. The first pathway demonstrated a transition from naïve T cells to Th17 cells, eventually differentiating into Tregs (Figure 5e). Early in ESCC, Th17 cells dominated immune regulation, activating neutrophils and macrophages. However, as the cancer progressed, the emergence of Tregs mitigated these pro-inflammatory effects, contributing to a predominantly immunosuppressive state [37]. The second pathway involved differentiation into *KLRB1*^+^ Th17 cells, emphasizing the pivotal role of Th17 cells in immune modulation during early disease stages [38].

To delve into regulatory mechanisms mediated by mpTFs during *CD4*^+^ T-cell differentiation, 203 T cell-related mpTFs were identified by screening TFs active in T cells across all cancer progression stages. Enrichment analysis identified mpTFs associated with two major pseudotime trajectories of *CD4*^+^ T-cell differentiation (Figure 5f and Figure S2). During the initial stages of cell differentiation, GABPβ2 regulated T cell development by promoting early lymphocyte generation, which was crucial for *CD4*^+^ T cell activation and proliferation. BACH2 preserved naïve T cell states by suppressing effector memory gene expression [39]. NFKB1, enriched in multiple differentiation phases, regulates T cell activation, proliferation, and functional maintenance. Its involvement highlighted its critical role in promoting immune responses during early differentiation stages and later contributing to immune homeostasis [40]. Furthermore, BATF and IRF4 facilitated Th17 differentiation by regulating IL-17-associated gene expression, crucial for pro-inflammatory responses and anti-tumor immunity [41]. VHL was closely associated with the differentiation of *FOXP3*^+^ Tregs and *TNFRSF18*^+^ Tregs. Through modulating the HIF-1α pathway, VHL was critical for maintaining the suppressive function of *FOXP3*^+^ Tregs, further promoting immune tolerance within the TME (Figure 5g) [42].

**Figure 6:**
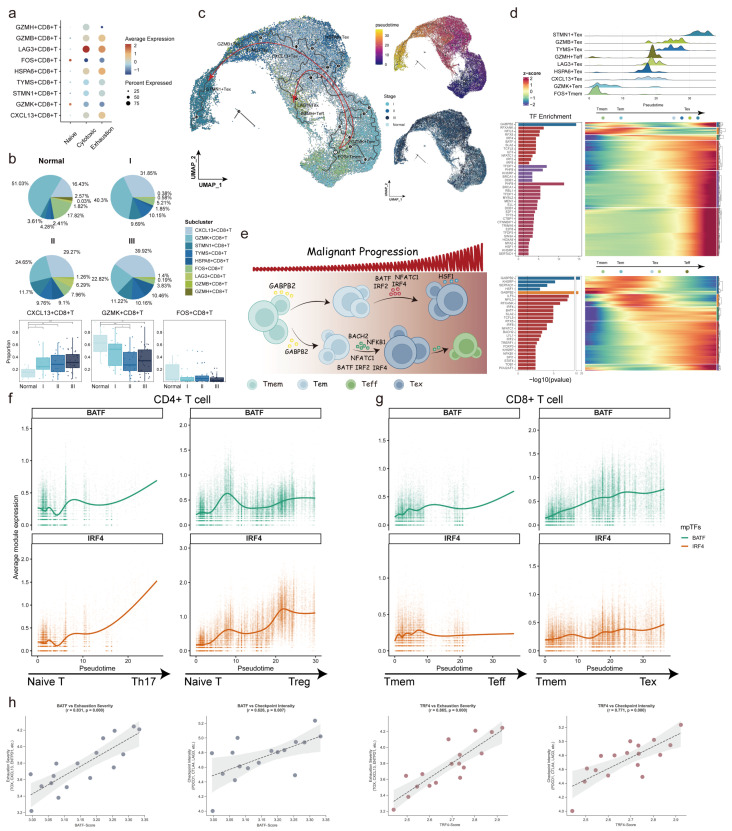
T cell-related mpTFs in altered *CD8*^+^ T cell fate. (**a**) Bubble plot showing average expression of various signatures in *CD8*^+^ T cell populations. (**b**) Pie charts showing proportions of *CD8*^+^ T subclusters in each disease stage (**top**), and box plots showing proportions of *CXCL13*+, *GZMK*+, and *FOS*+*CD8*^+^ T in samples from different stages (**bottom**). (**c**) UMAP plots showing the pseudotemporal trajectory of *CD8*^+^ T subclusters. (**d**) Heatmap showing the dynamic changes in gene expression associated with *CD8*^+^ T subclusters in Tmem to Tex cell trajectory and in Tmem to Teff cell trajectory, respectively. The density plot of the distribution of *CD8*^+^ T subclusters along the pseudotime is shown (**top**). Bar plot showing the enrichment of T cell-related mpTFs in genes associated with the two *CD8*^+^ T cell trajectories, respectively. (**e**) A graphical overview of mpTFs mediating *CD8*^+^ T cell fate transition. Arrows indicate inferred differentiation trajectories and regulatory transitions during malignant progression. (**f**,**g**) Dynamic activation patterns of BATF/IRF4-associated transcriptional programs along *CD4*^+^ T-cell differentiation trajectories and *CD8*^+^ T-cell exhaustion/differentiation trajectories. (**h**) External validation of the association between BATF/IRF4 activity and T cell dysfunction-associated programs in an independent pan-cancer T cell bulk transcriptomic dataset.

*CD8*^+^ T cells exhibited diverse functional states. *GZMH*^+^*CD8*^+^ T cells displayed effector T cell (Teff) characteristics, marked by cytotoxic activity through effector molecules like *GZMH* and transcription factors such as *HOPX*, which drove terminal effector differentiation. *GZMK*^+^*CD8*^+^ T cells, representing effector memory T cells (Tem), contributed to rapid immune responses upon antigen re-exposure, whereas *FOS*^+^*CD8*^+^ T cells, as long-term memory T cells (Tmem), played a key role in maintaining immune surveillance and providing durable protection against malignancy. However, *CD8*^+^ memory T cell populations declined as cancer progressed, giving way to exhausted T cells (Tex), characterized by high expression of immune checkpoint molecules like *LAG3* and *PDCD1* (Figure 6a,b). Pseudotime trajectory analysis revealed two major differentiation pathways in *CD8*^+^ T cells (Figure 6c). The first pathway traced the differentiation from Tmem and Tem cells to Tex cells, driven by sustained antigen stimulation and increasing immunosuppression [43,44]. The second pathway showed the differentiation from Tmem and Tem cells to Teff cells, with Tex cells serving as intermediates. TFs such as TBX21 (T-bet) promoted the differentiation from Tex to Teff cells by enhancing cytotoxic molecule gene expression while suppressing exhaustion programs (Figure 6d) [45].

Furthermore, we explored the influence of T cell-related mpTFs on *CD8*^+^ T-cell differentiation. GABPβ2 also participated in the early regulation of *CD8*^+^ T-cell differentiation trajectories, likely initiating the differentiation process by influencing the transcriptional programs in the initial cell population. During the transition from Tmem cells to Tex cells, IRF2 was critical in Tex cell functionality, upregulating exhaustion-associated genes [46]. Notably, BATF and IRF4, which regulated both *CD4*^+^ and *CD8*^+^ T-cell differentiation, activated effector molecule expression, such as *GZMA*, during Teff differentiation while suppressing exhaustion. Conversely, during the transition to Tex cells, these TFs cooperatively enhanced exhaustion-related transcriptional signatures, demonstrating their stage-specific regulatory roles in T cell fate determination. Additionally, TFs such as NFKB1 and BACH2 showed enrichment in the differentiation branch from Tmem cells to Teff cells, suggesting their unique roles in regulating Teff cell differentiation. NFKB1, a central regulator, promoted Tex cell formation by activating the NF-κB signaling pathway and exhaustion-related genes, underscoring its role in dictating the fate of *CD8*^+^ T cells under chronic stimulation (Figure 6e) [47].

To further validate the potential regulatory roles of BATF and IRF4 in T cell state remodeling, we performed additional trajectory-associated analyses in both *CD4*^+^ and *CD8*^+^ T cells. In *CD4*^+^ T cells, BATF/IRF4-associated transcriptional activity exhibited dynamic increases along the naïve-to-Th17 and naïve-to-Treg differentiation trajectories, suggesting their involvement in lineage-specific T cell state transitions (Figure 6f). Similarly, in *CD8*^+^ T cells, BATF/IRF4-associated programs progressively increased along the Tmem/Tem-to-Tex differentiation trajectory, whereas relatively weaker activation patterns were observed during Teff differentiation (Figure 6g). Furthermore, external validation using an independent pan-cancer T cell bulk transcriptomic dataset (GSE212697) demonstrated consistent positive associations between BATF/IRF4 activity and exhaustion/checkpoint-associated signatures (Figure 6h). Together, these findings provide additional computational evidence supporting the involvement of BATF and IRF4 in T-cell differentiation, exhaustion-associated state transitions, and immune dysregulation during ESCC progression. In summary, mpTFs played pivotal roles in regulating T-cell differentiation within the ESCC TME.

### 2.5. Promoting Immunosuppressive Microenvironment Formation by mpTFs

**Figure 7:**
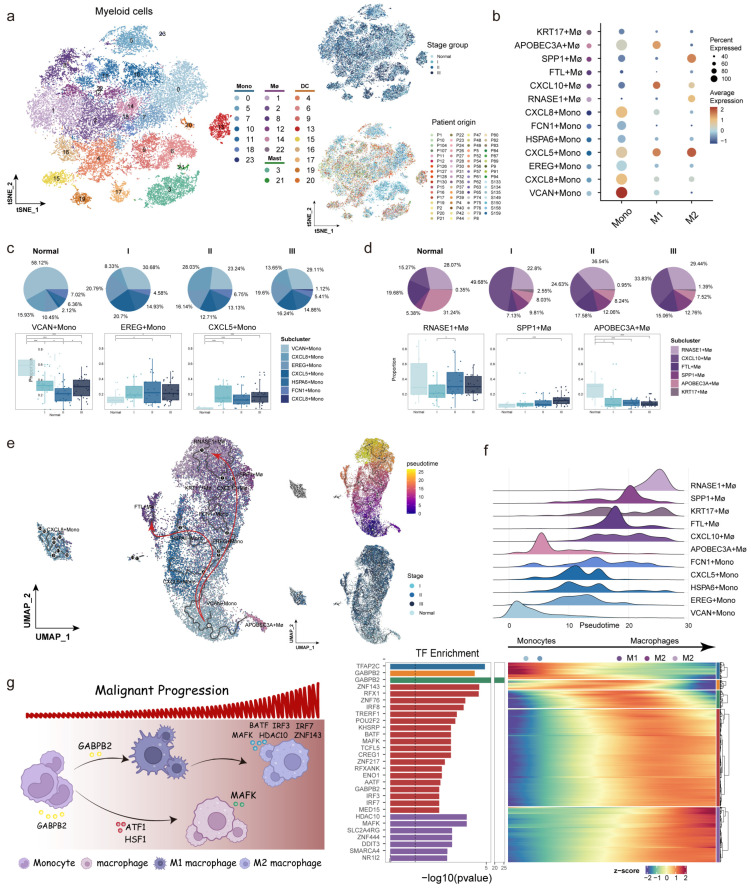
Macrophage phenotype changes affected by myeloid cell-related mpTFs. (**a**) tSNE plots showing 24 subclusters of myeloid cells, colored by the subcluster, disease stage, and patient origin. (**b**) Bubble plot showing average expression of monocyte, M1, and M2 signatures. (**c**) Pie charts showing proportions of monocyte subclusters in each disease stage, with box plots showing proportions of *VCAN*+, *EREG*+, and *CXCL5*+Mono in samples from different stages. Statistical significance was calculated by paired *t*-test. (**d**) Pie charts showing proportions of macrophage subclusters in each disease stage, with box plots showing proportions of *RNASE1*+, *SPP1*+, and *APOBEC3A*+Mø in samples from different stages. (**e**) UMAP plots showing the pseudotemporal trajectory of monocyte and macrophage subclusters, with red arrows showing trajectory direction, colored by subclusters (**left**) and stages (**bottom right**). Pseudotime coloring from purple to yellow (**top right**). (**f**) Heatmap showing the dynamic changes in gene expression associated with monocyte and macrophage subclusters in monocyte to M2 macrophage trajectory, with the density plot of the distribution of monocyte and macrophage subclusters along the pseudotime is shown (**top**). Bar plot showing the enrichment of myeloid cell-related MpTF in genes associated with the myeloid cell trajectory. (**g**) A graphical overview of mpTFs affecting phenotypic changes in myeloid cells.

Myeloid cells are integral components of the ESCC TME, playing crucial roles in immune regulation, inflammation, and tumor progression. A total of 34,553 myeloid cells were extracted, revealing 24 subclusters encompassing four cell populations: monocytes (*VCAN*, *S100A9*, and *LYZ*), macrophages (*RNASE1*, *APOE*, and *CXCL10*), dendritic cells (*CD1C*, *GNLY*, and *CCL22*), and mast cells (*TPSAB1* and *TPSB2*) (Figure 7a,b and S3a). Monocytes are vital innate immune cells that contribute to antigen presentation and pro-inflammatory responses, particularly in early tumor immunity [48]. *VCAN*^+^ Mono and *EREG*^+^ Mono displayed divergent roles. *VCAN*^+^ Mono, enriched in adjacent normal tissues, supported tissue repair and inflammation regulation. However, their proportions declined as ESCC progressed, likely due to immunosuppressive factors like IL-10 and TGF-β in the TME (Figure 7c). Conversely, *EREG*^+^ Mono increased in tumors, suggesting their involvement in tumor-promoting cytokine secretion. *CXCL5*^+^ Mono, with elevated tumor proportions, likely influenced progression by recruiting neutrophils and creating a pro-inflammatory environment. Tumor-associated macrophages (TAMs) exhibited dynamic phenotypic transitions. Pro-inflammatory M1 macrophages (*CXCL10*^+^ and *APOBEC3A*^+^ Mø) were enriched in early stages, displaying tumor-suppressive properties through cytokine secretion and antigen presentation. However, their proportions decreased as ESCC progressed, coinciding with increased M2 macrophages (*SPP1*^+^ and *RNASE1*^+^ Mø) that promoted immune evasion, angiogenesis, and tumor growth by secreting IL-10 and TGF-β [49] (Figure 7d).

Pseudotime trajectory analysis revealed the progressive differentiation of monocytes into diverse macrophage phenotypes during ESCC progression (Figure 7e,f). Early in differentiation, monocytes dominated the TME immune response. Pro-inflammatory M1 macrophages, particularly *CXCL10*^+^ Mø, emerged during early tumor stages, driving antigen presentation and effector immune activation. As the tumor advanced, M1 macrophages transitioned to anti-inflammatory M2 macrophages under tumor-derived signals, underscoring the immunosuppressive remodeling of the TME [50,51]. Interestingly, *FTL*^+^ Mø, enriched at differentiation endpoints, lacked typical M1/M2 markers but expressed *MMP12*, a gene closely related to M2 macrophage activation in ESCC (Figure S3b) [52], indicating a potential to further differentiate into anti-inflammatory M2 macrophages.

To further investigate the regulatory mechanisms mediated by mpTFs during macrophage phenotype transitions, we identified 231 myeloid-related mpTFs and analyzed their regulatory roles along the two primary differentiation trajectories of myeloid cells. (Figure 7f and Figure S3). GABPB2 was implicated in early monocyte differentiation, consistent with its established role in myeloid development [53]. Similarly, we observed that MAFK regulated macrophages across different differentiation trajectories. Although MAFK did not directly influence the inflammatory tumor environment, it promoted inflammatory responses by modulating NF-κB activation [54]. During the differentiation of monocytes into M2 macrophages, IRF3 and IRF7 were identified as key regulators of anti-inflammatory M2 macrophage functions. While IRF3 was typically associated with M1 macrophage activation, it could induce M2 polarization in tumors via HSP90α secreted by tumor-associated fibroblasts [55]. IRF7, conversely, inhibited tumor progression by reversing the ability of M2 macrophages to induce cell proliferation and migration [56]. Additionally, HDAC10, potentially regulated by NSUN6-mediated m5C methylation, recruited M2 macrophages, highlighting a late-stage tumor adaptation mechanism [57]. HSF1 emerged as a central regulator of monocyte-to-macrophage differentiation, inducing macrophage phenotypes via SPI1/PU.1 co-expression, a critical driver of macrophage lineage commitment (Figure 7g) [58].

In summary, we elucidated the complex roles of myeloid-related TFs in tumor immune evasion and malignancy progression, providing important insights into the inflammatory microenvironment of ESCC and informing potential therapeutic strategies targeting TF-regulated pathways.

### 2.6. mpTFs as Prognostic Indicators for ESCC Patients

**Figure 8:**
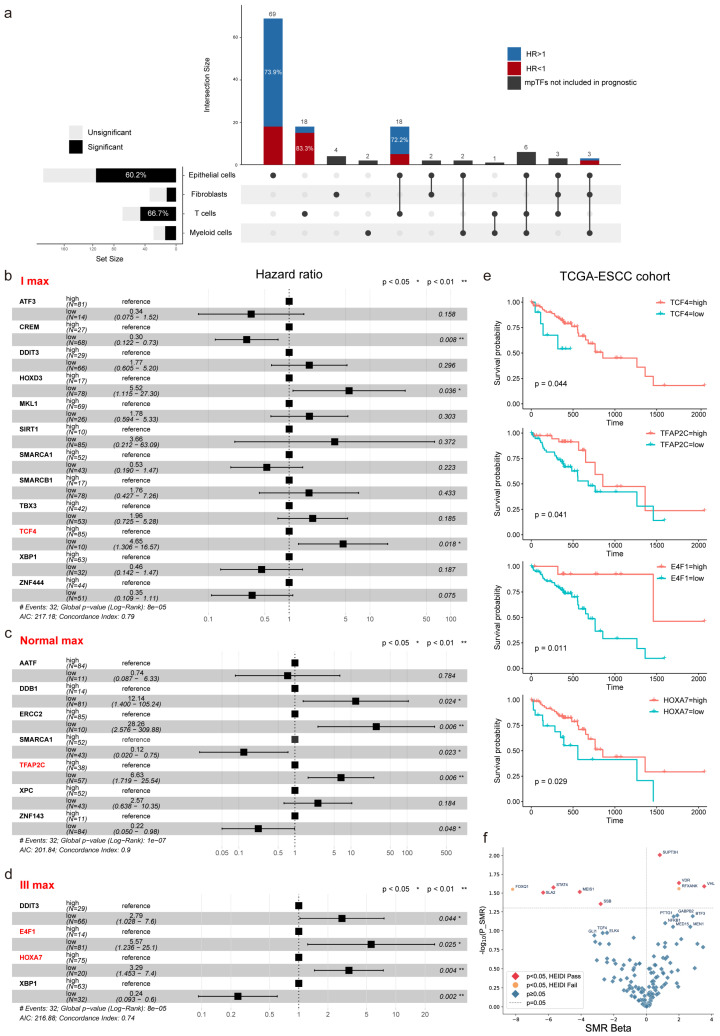
Malignant epithelial EMT and fibroblast mpTFs correlated with patient survival. (**a**) Upset plot showing overlap of prognostic-related mpTFs across epithelial cells, fibroblasts, T cells, and myeloid cells. Cell type–specific and epithelial–T shared mpTFs were classified by hazard ratio (HR > 1, risk; HR < 1, protective). Left black bars indicate the proportion of significant mpTFs, and top black bars indicate mpTFs not included in the prognostic (**b**,**d**) Prognostic values of twelve mpTFs with the I Max characteristic in EMT (**b**), seven mpTFs with the Normal characteristic (**c**), and four mpTFs with the III Max characteristic (**d**) in fibroblasts, using data from 95 patients in the TCGA-ESCC cohort. Forest plots display hazard ratios and survival levels derived from Cox regression analysis. Statistical significance is indicated for *p*  <  0.05. (**e**) Kaplan–Meier survival curves showing overall survival for ESCC patients with high expression of TCF4, TFAP2C, E4F1, and HOXA7, respectively. (**f**) SMR analysis of prognosis-associated mpTFs using the ESCA overall survival GWAS dataset. Red points indicate genes passing both SMR significance and HEIDI tests, suggesting putative causal associations with patient survival.

To explore the impact of cell type-related mpTFs on ESCC prognosis, we evaluated the influence of mpTFs on patient survival across cell types using Cox regression and Kaplan–Meier survival analyses (Figure 8). Based on the reproducibility of mpTFs across cell types, we further explored the cell-type-specific and shared mpTFs. Over 60% of malignant epithelial and T cell-related mpTFs were associated with ESCC prognosis (Figure 8a). Most malignant epithelial-specific mpTFs (>70%) were protective, whereas the T cell-specific mpTFs tend to be risk factors for ESCC (>80%). Notably, the malignant epithelial cell-specific mpTF TCF4, which regulated EMT in stage I ESCC, was significantly linked to patient survival (Figure 8b–e). This implied that TCF4 might contribute to invasive phenotypes through EMT-related gene expression, making it a potential prognostic marker for early-stage ESCC. Moreover, we found that there were more interactions between malignant epithelial cells and other cell types in the TME. Most of these mpTFs were associated with suppressive effects on tumor initiation and progression by modulating signaling pathways and biological processes, such as cell proliferation and metabolism (Figures S4–S11). Some mpTFs, such as IRF4 and BATF, are associated with poor prognosis (Figures S4a,b,d and S7). They influenced metabolic processes in advanced malignant epithelial cells and regulated T-cell differentiation and exhaustion, acting synergistically to foster a highly immunosuppressive TME that promoted disease progression. Additionally, we identified three mpTFs shared between malignant epithelial cells, fibroblasts, and myeloid cells, including the protective factor TFAP2C and the risk factors ZNF76 and ZNF143. TFAP2C was expressed in NFs and csCAFs and regulated cell proliferation and development in the mid-stage of malignant epithelial cells. It also regulated monocyte differentiation, with high expression linked to better survival outcomes. This suggested that TFAP2C stabilized the functional states of CAFs and monocytes, contributing to a less aggressive TME during early-stage ESCC, acting as a protective factor. In contrast, ZNF143 contributed to an immunosuppressive microenvironment by upregulating IL-10 and VEGF. This regulatory activity facilitated macrophage polarization towards the immunosuppressive M2 phenotype and activated the hypoxic pathway in malignant epithelial cells, correlating with poor prognosis in ESCC. In summary, mpTFs shared between these cell types interacted at various stages in the TME to influence ESCC progression.

In parallel with the prognostic analyses, we further performed summary-data-based Mendelian randomization (SMR) analysis using the Esophageal Carcinoma overall survival GWAS dataset integrated within the SUMMER platform to investigate genetically inferred associations between mpTF expression and ESCC patient survival (Figure 8f and Table S5) [59,60]. This analysis identified seven mpTFs showing significant putative causal associations with ESCC prognosis, providing additional genetic evidence linking mpTF dysregulation to disease progression and clinical outcomes. Notably, several mpTFs identified through the single-cell regulatory network analysis were consistently supported in the SMR framework, further highlighting their potential importance during ESCC progression. These findings suggest that certain mpTFs may not only correlate with patient survival but may also participate in regulatory processes influencing disease progression. Collectively, these results highlight mpTFs as potential prognostic biomarkers and promising targets for clinical molecular subtyping and precision therapies in ESCC.

## 3. Discussion

The highly heterogeneous cellular and molecular nature of ESCC, coupled with the absence of stage-specific biomarkers, complicated the development of effective therapies. Although recent metabolomic, genomic and proteomic studies have provided insights into ESCC carcinogenesis and progression [61,62], they have struggled to resolve the roles of major cellular players at various stages of cancer progression or elucidate the key molecular mechanism, particularly the regulatory functions of TFs at the single-cell level. Here, we systematically constructed a comprehensive single-cell atlas focused on TF regulatory networks in ESCC, and uncovered dynamic transcriptional programs associated with tumor progression, stromal remodeling, and immune dysfunction within the TME. Our analyses revealed extensive transcriptional reprogramming across malignant epithelial, stromal, and immune compartments during ESCC progression. Among malignant epithelial cells, EMT-associated programs emerged as prominent features during early-stage progression, suggesting that transcriptional plasticity may contribute to invasive phenotypes at relatively early disease stages. Notably, TCF4 was strongly associated with EMT-related programs and poor prognosis. Additional virtual knockout analyses further supported the potential involvement of TCF4 in EMT-, migration-, and extracellular matrix remodeling-associated transcriptional programs, highlighting a possible regulatory role of TCF4 in early ESCC invasive progression.

In stromal compartments, fibroblast-associated TF programs were linked to extracellular matrix remodeling, inflammatory regulation, and antigen presentation, suggesting that transcriptional reprogramming of CAFs may actively shape the immunosuppressive microenvironment during ESCC progression. Similarly, myeloid cell analyses revealed progressive macrophage polarization toward immunosuppressive M2-like states, highlighting dynamic inflammatory remodeling within the TME. Several TF-associated regulatory programs were linked to monocyte-to-macrophage differentiation and macrophage functional states, suggesting potential roles of TF-mediated myeloid remodeling in immune suppression and tumor-promoting inflammatory environments.

A major finding of this study was the dynamic immune remodeling observed during ESCC progression. *CD4*^+^ and *CD8*^+^ T cells progressively transitioned toward immunosuppressive and dysfunctional states under chronic stimulation within the TME. TFs such as BATF, IRF4, BACH2, and HSF1 were associated with these differentiation trajectories and exhaustion-associated programs, suggesting that TF-mediated transcriptional reprogramming may contribute to immune evasion during ESCC progression. Additional trajectory-associated and external transcriptomic validation analyses further supported the involvement of BATF/IRF4 in T cell state remodeling and dysfunction-associated programs.

We further identified multiple prognosis-associated mpTFs across malignant, stromal, and immune cell populations, highlighting extensive transcellular regulatory interactions within the ESCC microenvironment. Although these associations were primarily correlational, we incorporated SMR-based causal inference analysis to provide additional genetic evidence [59,60,63]. Seven mpTFs exhibited putative causal associations with patient outcomes, further supporting their potential involvement in ESCC progression and clinical prognosis. Nevertheless, these findings should be interpreted cautiously due to the limited availability of ESCC-specific survival GWAS datasets.

Several limitations of the present study should be acknowledged. Although we incorporated multiple computational validation strategies, including virtual knockout analysis, trajectory-associated functional validation, external transcriptomic validation, and SMR-based causal inference analysis, the regulatory functions of several mpTFs still require direct experimental validation in vitro and in vivo. Future studies integrating multi-omics approaches and functional perturbation experiments will help further clarify the mechanistic roles of these mpTFs in tumor evolution and immune remodeling.

Collectively, our study provides a single-cell resolution framework for understanding TF-driven regulatory programs during ESCC progression, highlighting dynamic interactions between malignant, stromal, and immune compartments and offering potential directions for biomarker discovery and precision therapeutic intervention.

## 4. Materials and Methods

### 4.1. scRNA-Seq Data Collection from ESCC Samples

We collected two scRNA-Seq datasets from GEO (https://www.ncbi.nlm.nih.gov/geo/, accessed on 30 May 2025) for ESCC patients, which included both tumor tissues and adjacent normal tissues. A total of 67 patients were involved, with 67 tumor samples and 11 normal tissue samples. Among the tumor samples, 16 were from stage I, 21 from stage II, and 30 from stage III. Detailed information on the samples is provided in Table S1.

### 4.2. scRNA-Seq Data Processing

The scRNA-seq data were analyzed using the R package Seurat (version 4.4.0) [64]. A total of 204,831 cells were used for downstream analyses after filtering out low-quality cells (those expressing fewer than 200 or more than 4000 genes, and with a mitochondrial genome proportion greater than 5%).

The “FindVariableFeatures” function was used to identify the top 2000 highly variable genes for principal component analysis, followed by dimensionality reduction using “RunPCA” and batch effect correction with “RunHarmony”. The “FindClusters” function (resolution set to 0.7) was used to identify different subclusters, which were annotated into seven main cell types based on classical markers and previously reported characteristic gene sets [16,51]: epithelial cells (*EPCAM*, *SFN*, *KRT5*, *KRT14*, *SPRR3*), fibroblasts (*FN1*, *DCN*, *COL1A1*, *COL1A2*, *COL3A1*, *COL6A1*), pericytes (*RGS5*, *MCAM*, *ACTA2*), endothelial cells (*VWF*, *PECAM1*, *ENG*, *CDH5*, *RAMP2*), T cells (*CD2*, *CD3D*, *CD3E*, *CD3G*, *CXCL13*, *CCL5*), B cells (*CD79A*, *JCHAIN*, *MZB1*, *IGKC*), and myeloid cells (*CD68*, *LYZ*, *CD14*).

To identify specific cell subtypes, each major cell type was separately normalized, reduced in dimensionality, and clustered. The “FindAllMarkers” function was used to identify marker genes for each subcluster, and differential analysis was performed using the Wilcoxon rank-sum test with criteria set to pct = 0.25, logfc.threshold = 0.25, and only.pos = TRUE. Detailed annotation of major cell subtypes was achieved by integrating differentially expressed genes, the CellMarker database [65], and prior studies [16,51].

### 4.3. Identification of Malignant Progression-Associated Transcription Factors Across Cell Types

We designed a computational framework for the systematic identification of malignant progression-associated transcription factors across cell types, which consists of three components:

(i) Gene regulatory network construction. We collected and integrated the transcription factor target gene dataset from the TRRUST database [66] and protein–protein interaction (PPI) network data from the HPRD database (Human Protein Reference Database) [67]. We then used the R package igraph (https://github.com/igraph/rigraph, accessed on 12 June 2025) to construct a comprehensive gene regulatory network.

(ii) Identifying transcription factor-associated gene modules using the random walk algorithm. Based on our previous study [68], a collection of transcription factor significantly related genes was systematically mined from the gene regulatory network based on the random walk algorithm. Specifically, we treated a single transcription factor as a seed node, assigning the seed transcription factor a weight of 1 and the rest of the transcription factors a weight of 0. The influence score of this seed transcription factor on genes in the network was assessed based on the neighbor-joining matrix of the gene regulatory network. We then sorted all the genes from high to low according to the influence score, selecting the top 50 genes as the related gene set for that transcription factor. Next, we mined significant transcription factor–gene module relationships. Specifically, we considered a transcription factor–gene module relationship to be significantly related if the following two conditions were met: (1) there was a stable network connection between the transcription factor and the gene module, and (2) the transcription factor-associated gene module significantly overlapped with the corresponding target genes of the transcription factor. We first constructed a gene regulatory subnetwork for the transcription factor based on the transcription factor–gene module relationship and calculated the connectivity of this network to measure its stability. We considered the transcription factor gene regulatory subnetwork with connectivity greater than 5 to be stable. To assess the significance of the overlap between the transcription factor-associated gene modules and the corresponding target genes, we calculated the p-value using the hypergeometric test, as follows:

$$p=1-\sum_{k=0}^{x-1}\frac{\left(\begin{array}{c} M\\ k \end{array}\right)\left(\begin{array}{c} N-M\\ n-k \end{array}\right)}{\left(\begin{array}{c} N\\ n \end{array}\right)}$$

For transcription factor i and its gene module j, where N represents the total number of genes in the integrated gene regulatory network except the transcription factor, M is the number of target genes corresponding to transcription factor i in the TRRUST dataset, n is the number of genes in gene module j, and x is the number of genes overlapped between M and n, we retained the transcription factor and its gene module if *p* < 0.05.

Finally, all transcription factors were traversed and all significant transcription factor-related gene modules were mined.

(iii) Identification of malignant progression-associated transcription factors. We integrated transcription factor-related gene modules with scRNA-Seq data to calculate the transcription factor activity in seven cell types spanning four disease stages. Referring to methods from an existing study [69], we assessed the distribution differences in the relative gene expression levels of transcription factor-related gene modules (gene sets) across different cell types to characterize transcription factor activity. The steps for calculating transcription factor-related gene module activity were as follows:

First, the average expression of gene i in cell type j was calculated as $E_{i,j}$:

$$E_{i,j}=\frac{\sum_{k=1}^{n_{j}}g_{i,k}}{n_{j}},i\in 1\text{...}M,j\in 1\text{...}N$$

where $n_{j}$ is the number of cells in cell type j, $g_{i,k}$ is the expression of gene i in that cell type, M is the number of genes in the module, and N is the number of cell types.

Next, the relative expression of gene i in cell type j is calculated as $R_{i,j}$:

$$R_{i,j}=\frac{E_{i,j}}{\frac{1}{N}\sum_{j}^{N}E_{i,j}}$$

Finally, the activity score $M_{t,j}$ of the transcription factor t-related gene module in cell type j was calculated:

$$M_{t,j}=\frac{\sum_{i=1}^{m_{t}}w_{i}\times R_{i,j}}{\sum_{i=1}^{m_{t}}w_{i}}$$

where $m_{t}$ is the number of genes in the transcription factor t-associated gene module, and the weight factor $w_{i}$ is the reciprocal of the number of transcription factor-associated gene modules containing the *i*th gene. According to an existing study [69], genes with relative expression higher than threefold or lower than one-third were excluded, thus removing outliers in each transcription factor-related gene module. The statistical significance *p* of the transcription factor-related gene modules in cell types was then assessed by randomizing the cell type labels in the scRNA dataset 5000 times. Finally, we compared the activity scores of transcription factor-related gene modules in the randomized dataset with those in the original (non-randomized) dataset. The proportion of transcription factor activities in the randomized dataset that were either greater than $M_{t,j}$ ($M_{t,j}$ > 1) or less than $M_{t,j}$ ($M_{t,j}$ < 1) was calculated and defined as the significance *p*. We considered the transcription factor activities with *p* < 0.05 to be significantly higher (or lower) than the average.

### 4.4. Distinguishing Malignant Epithelial Cells Based on Copy Number Variations

The R package infercnv (https://github.com/broadinstitute/infercnv, accessed on 4 June 2025) was used to infer copy number variation (CNV) scores of epithelial cells in ESCC, with fibroblasts serving as the reference group for CNV score calculation. Genes with an average expression count below 0.1 in cells were filtered and denoised. After obtaining the score for each cell, the matrices corresponding to the reference fibroblasts and observed epithelial cells were extracted for K-means clustering. After removing the cells clustered together with the cells of the reference group, malignant epithelial cells were finally identified.

### 4.5. Identification of Gene Expression Programs in Malignant Epithelial Cells

We selected the count matrix of all malignant epithelial cells to reveal their malignant features using common non-negative matrix factorization (cNMF) (https://github.com/dylkot/cNMF, accessed on 8 June 2025) [70]. Different k values were chosen for different subclusters of malignant epithelial cells to ensure accuracy. The top 100 genes, ranked by their contribution scores to specific gene expression programs (GEPs), were selected as representative gene sets for each gene expression program. Finally, functional enrichment analysis was performed to annotate the identified gene expression programs using hallmark gene sets from the MSigDB database (https://www.gsea-msigdb.org/gsea/msigdb, accessed on 8 June 2025).

### 4.6. Functional Enrichment Analysis

We used the R packages org.Hs.eg.db (3.23.1) and clusterProfiler (4.20.0) to perform functional enrichment analyses for different cell subtypes and transcription factors. The “enrichGO” function was used to perform Gene Ontology (GO) enrichment analysis for cell subtype differential genes and transcription factor gene modules, with the criteria set to ont = “BP”, pvalueCutoff = 0.05, and qvalueCutoff = 0.05. The Gene Ontology Resource (http://www.geneontology.org, accessed on 8 June 2025) was used to further categorize the GO functions of transcription factor gene modules.

### 4.7. Virtual Knockout Experiment

A virtual knockout experiment was performed using the scTenifoldKnk (1.0.3) framework [27]. Early-stage malignant epithelial cells were extracted to construct reference gene regulatory networks (GRNs). Virtual knockout of target genes was performed by removing their regulatory influence, and perturbed networks were reconstructed. Differentially perturbed genes (DPGs) were identified based on topological differences between networks and used for downstream functional enrichment analysis.

### 4.8. Transcription Factor Correlation Analysis

For malignant epithelial cells, Gene Set Variation Analysis (GSVA) [71] was applied to calculate the score of all gene sets with contributions in GEPs. For fibroblasts, GSVA was applied to the set of genes with significantly high expression in fibroblast subtypes to calculate their scores. The correlation between transcription factor gene modules and malignant epithelial cell GEPs, as well as between transcription factor gene modules and fibroblast subtypes, was calculated using the “rcorr” function from the R package Hmisc. The default parameters were used, and correlation coefficients > 0.4 with *p* < 0.05 were considered significant.

### 4.9. Cell Differentiation Trajectory Construction

Cell differentiation trajectories were analyzed by the R package monocle3 to explore the differentiation process of various cell types [72]. First, the monocle3 object was created using the “new_cell_data_set” function, and the data were visualized using Uniform Manifold Approximation and Projection (UMAP) for dimensionality reduction. Subsequently, subclusters were partitioned using the “cluster_cells” function (default parameter) and the use_partition parameter was set to TRUE in the “learn_graph” function. Due to the independence of the partitions in which the two subclusters of CXCL8+ Mono were embedded, their trajectories were excluded from the subsequent analyses. Additionally, we defined subclusters primarily from adjacent normal tissues as “root cells” for pseudotime analysis. Differential genes in the trajectories were identified by the “graph_test” function and ranked according to their effect sizes on the trajectories. Genes with *q*_value < 0.05 and morans_I > 0.1 were considered significantly associated with the differentiation trajectories.

### 4.10. Transcription Factor Enrichment Analysis

To identify transcription factors related to the cell differentiation process, we used the “enricher” function from the R package clusterProfiler to enrich transcription factors. Genes related to the differentiation trajectory were defined as the background gene set, and transcription factor gene modules were enriched with the parameters pvalueCutoff = 0.05 and qvalueCutoff = 0.05.

### 4.11. Analysis of Survival

We obtained bulk RNA-seq data and corresponding clinical information for ESCC samples from existing studies [73,74]. To assess the impact of transcription factors on ESCC patient survival, GSVA was applied to calculate the combined expression values of transcription factor gene modules (gene sets). The “surv_cutpoint” function from the R package Survminer was used to classify patients into high and low expression groups, and Kaplan–Meier survival curves were plotted. Prognostic models were then constructed using a multivariate Cox proportional hazards model for the set of transcription factors across different cell types, and the results were visualized using the “ggforest” function.

### 4.12. Mendelian Randomization Analysis

To further evaluate the potential causal relationships between prognosis-associated mpTFs and overall survival (OS) in ESCC, summary-data-based Mendelian randomization (SMR) analysis was performed. Given the limited availability of ESCC-specific survival GWAS resources, esophageal cancer (ESCA) overall survival summary statistics were used as a surrogate dataset for causal inference analysis. Cis-eQTL data for transcription factor genes were obtained from the eQTLGen consortium, while ESCA OS GWAS summary statistics were retrieved from the SUMMER platform (UK Biobank cohort) [60]. For mpTFs with significant cis-eQTL signals, SMR analysis was conducted to assess the association between genetically predicted gene expression and patient survival outcomes, followed by the HEIDI test to exclude associations caused by linkage disequilibrium. Multiple testing correction was performed using both the FDR and Bonferroni methods. Transcription factors simultaneously meeting the SMR significance threshold, HEIDI test criteria, and prognostic significance were defined as candidate mpTFs with putative causal associations with patient survival.

## 5. Conclusions

In conclusion, our study provides a comprehensive single-cell transcription factor regulatory atlas of ESCC and reveals the dynamic cellular and molecular remodeling that occurs during disease progression. We identified marked heterogeneity among malignant epithelial cells, fibroblasts, T cells, and myeloid cells, and demonstrated that stage-dependent transcription factor programs shape tumor progression, stromal remodeling, immune differentiation, and immune suppression within the tumor microenvironment. Importantly, we uncovered both cell-type-specific and shared master prognostic transcription factors associated with ESCC progression and patient outcome, highlighting their potential value as biomarkers and therapeutic targets. Together, these findings deepen our understanding of the regulatory mechanisms underlying ESCC evolution and offer a valuable resource for stage-specific diagnosis, prognostic assessment, and precision treatment strategies.

## Data Availability

All data needed to evaluate the conclusions in the paper are present in the paper and/or the Supplementary Materials. All accession codes, unique identifiers, or web links for publicly available datasets are described in the paper. All codes are available upon reasonable request.

## Supplementary Materials

The following supporting information can be downloaded at: https://www.mdpi.com/article/10.3390/ijms27104433/s1.Figure S1: epithelial cell copy number inference and additional validation analyses; Figure S2: enrichment analysis of major pseudotime trajectories during *CD4*^+^ T-cell differentiation; Figure S3: identification and differentiation characteristics of myeloid cell subtypes during ESCC progression; Figure S4–S11: prognostic characteristics and survival analyses of mpTFs across different cell and function types; Table S1: sample information included in this study; Table S2: mpTF-associated gene regulatory modules; Table S3: gene sets associated with gene expression programs (GEPs); Table S4: mpTFs involved in stage-specific gene expression programs; Table S5: summary-data-based Mendelian randomization (SMR) analysis results.

## Abbreviations

The following abbreviations are used in this manuscript:

ESCC	Esophageal Squamous Cell Carcinoma
TF	Transcription Factor
scRNA-seq	Single-cell RNA sequencing
EMT	Epithelial–Mesenchymal Transition
TME	Tumor Microenvironment
GRN	Gene Regulatory Network
GEP	Gene Expression Program
tSNE	t-distributed Stochastic Neighbor Embedding
UMAP	Uniform Manifold Approximation and Projection
CNV	Copy Number Variation
ECM	Extracellular Matrix
NF	Normal fibroblast
CAF	Cancer-associated Fibroblast
csCAF	Complement-secreting CAF
meCAF	Metabolic CAF
mCAF	Matrix CAF
iCAF	Inflammatory CAF
adiCAF	Adipogenic CAF
apCAF	Antigen-presenting CAF
myCAF	Myofibroblast
pCAF	Proliferative CAF
cNMF	Common Non-negative Matrix Factorization
TAM	Tumor-associated Macrophages
Tex	Exhausted T cells
Tem	Effector Memory T cell
Tmem	Long-term Memory T cell
Teff	Effector T cell
GO	Gene Ontology
GSVA	Gene Set Variation Analysis
