# Supplementary figures and images for: Deciphering the Transcription Factor-Dominated Ecosystem During Esophageal Squamous Cell Carcinoma Progression at the Single-Cell Level
Source: Int J Mol Sci. 2026 May 15;27(10):4433. doi: 10.3390/ijms27104433 (PMC13206960; doi:10.3390/ijms27104433)

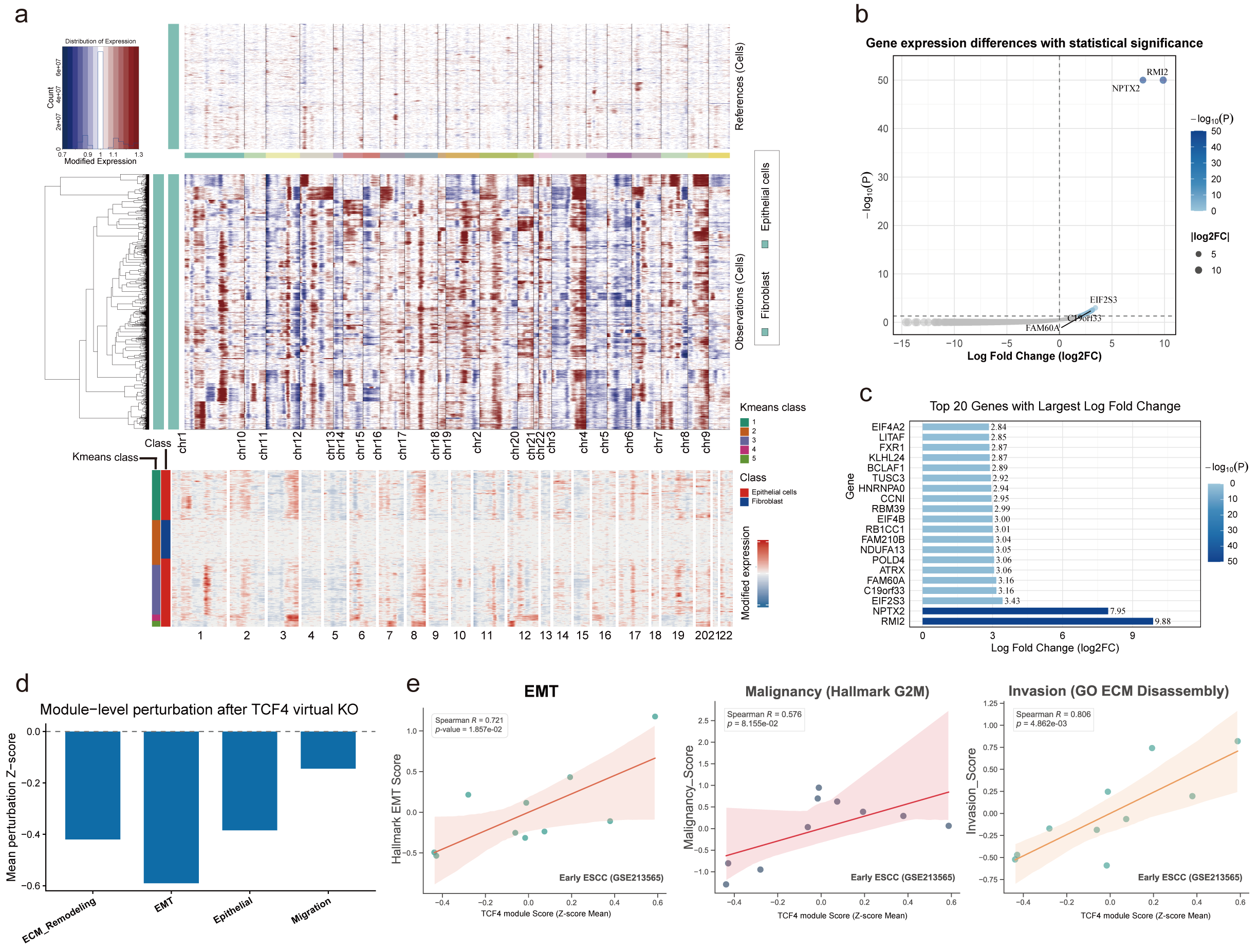

Supplement: Supplementary file 1 [file ijms-27-04433-s001.zip › Fig. S1.tif]

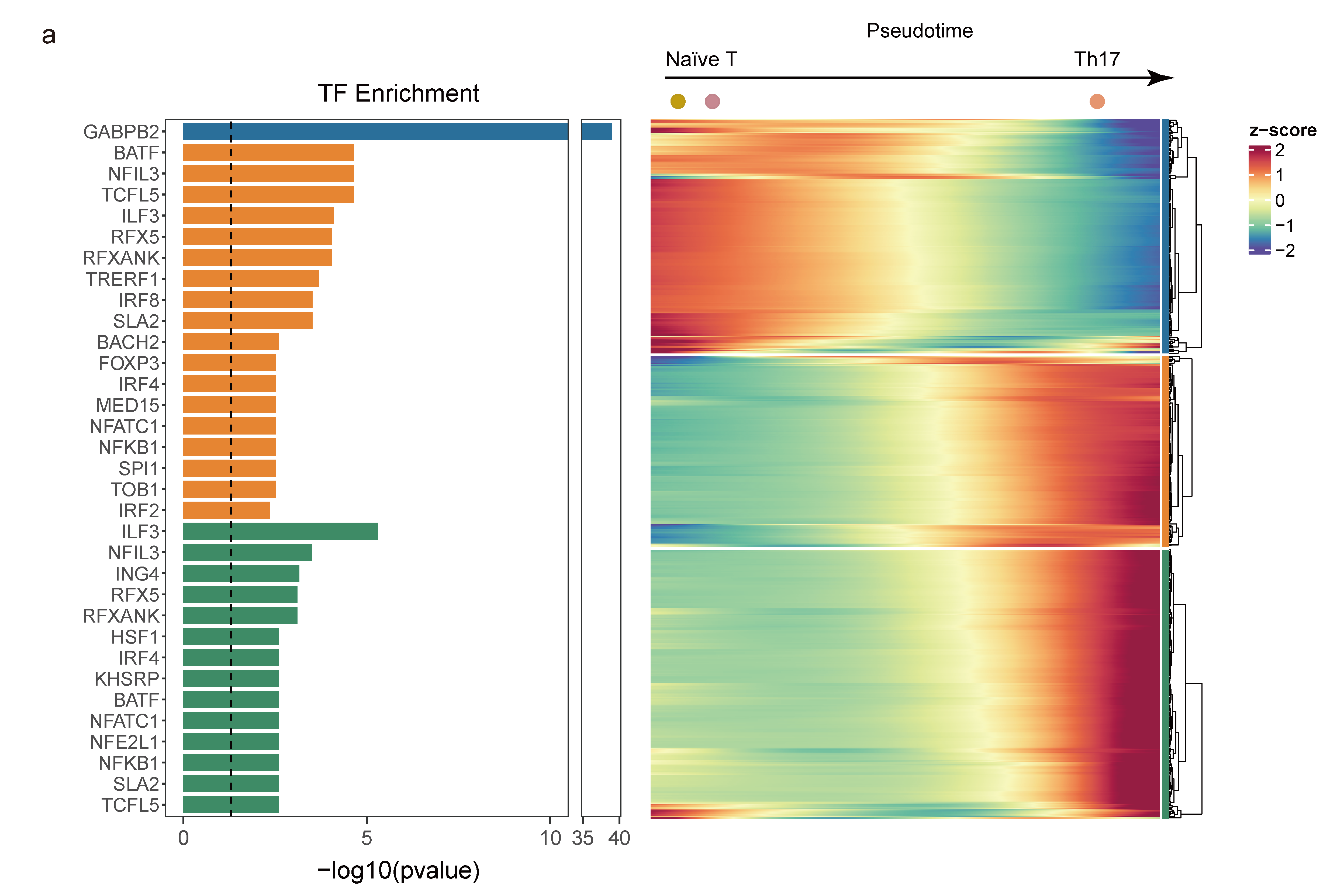

Supplement: Supplementary file 1 [file ijms-27-04433-s001.zip › Fig. S2.tif]

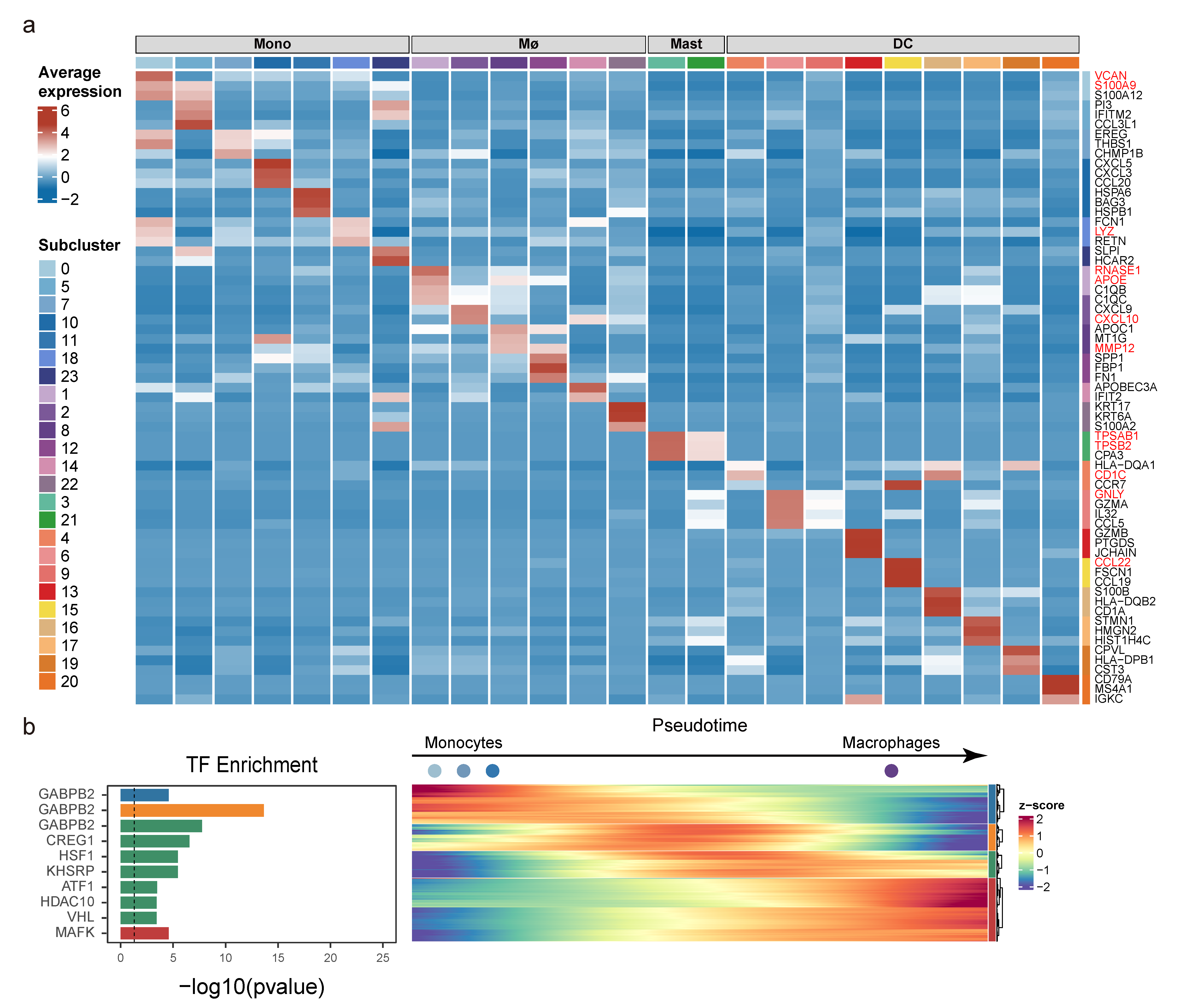

Supplement: Supplementary file 1 [file ijms-27-04433-s001.zip › Fig. S3.tif]

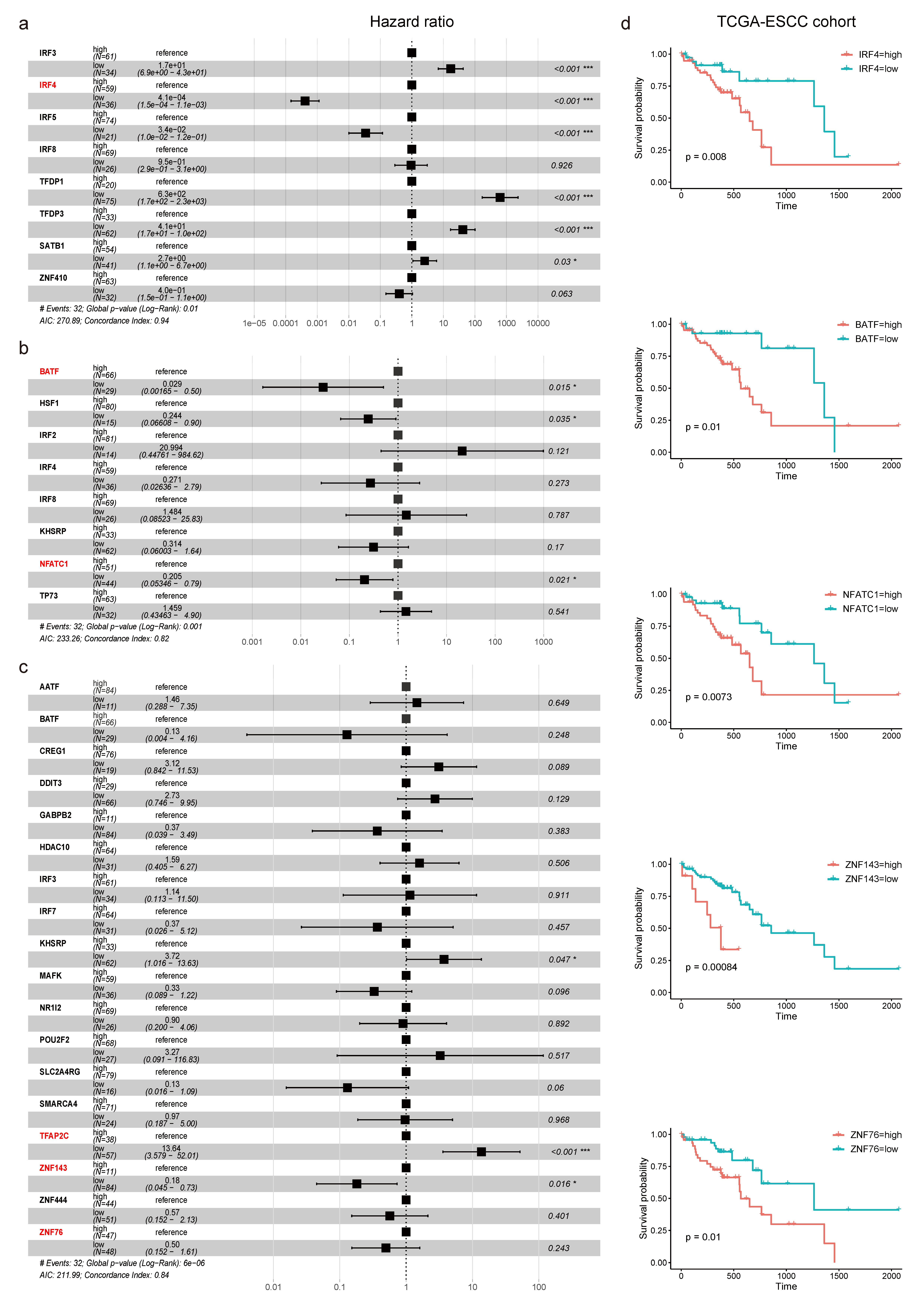

Supplement: Supplementary file 1 [file ijms-27-04433-s001.zip › Fig. S4.tif]

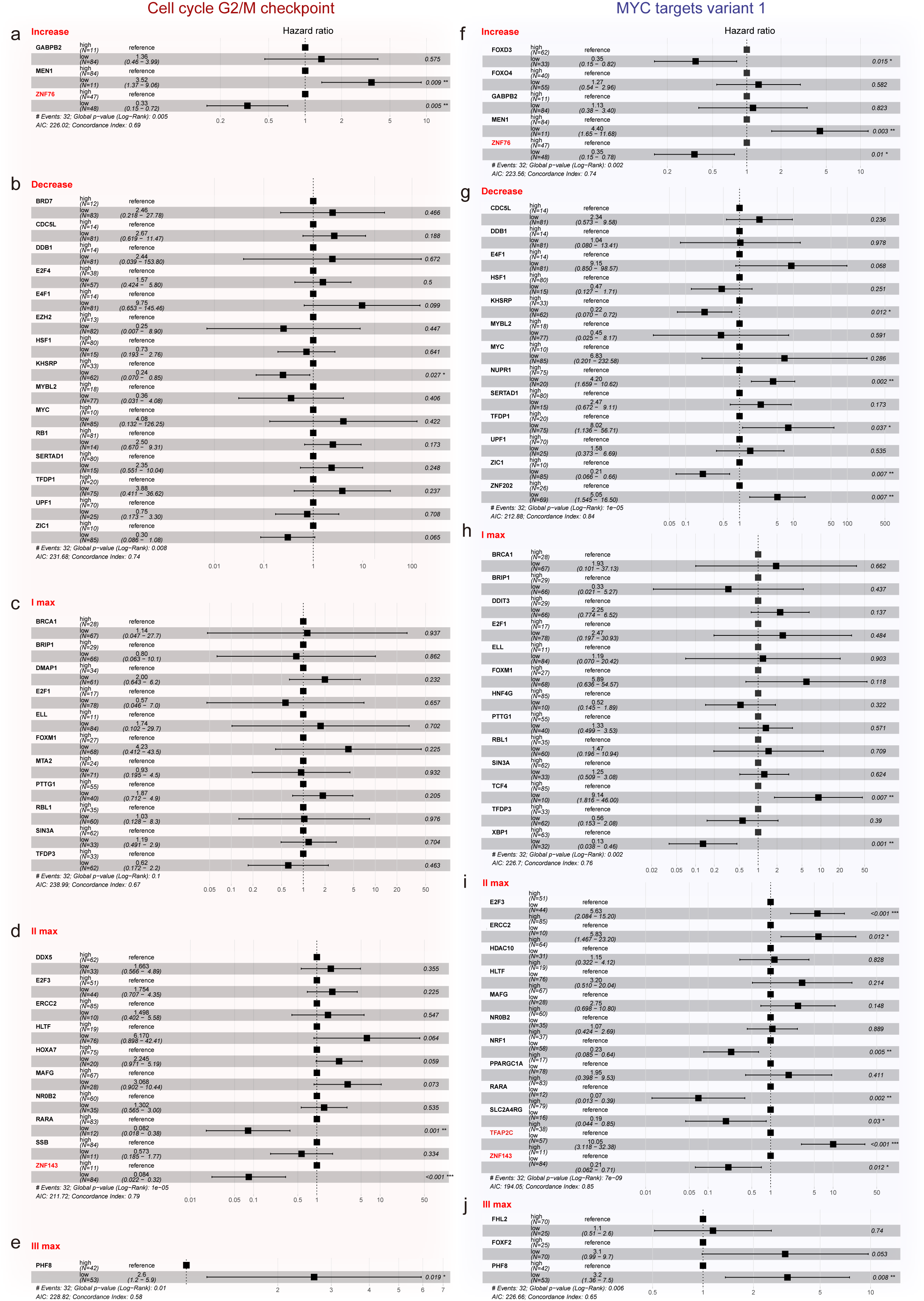

Supplement: Supplementary file 1 [file ijms-27-04433-s001.zip › Fig. S5.tif]

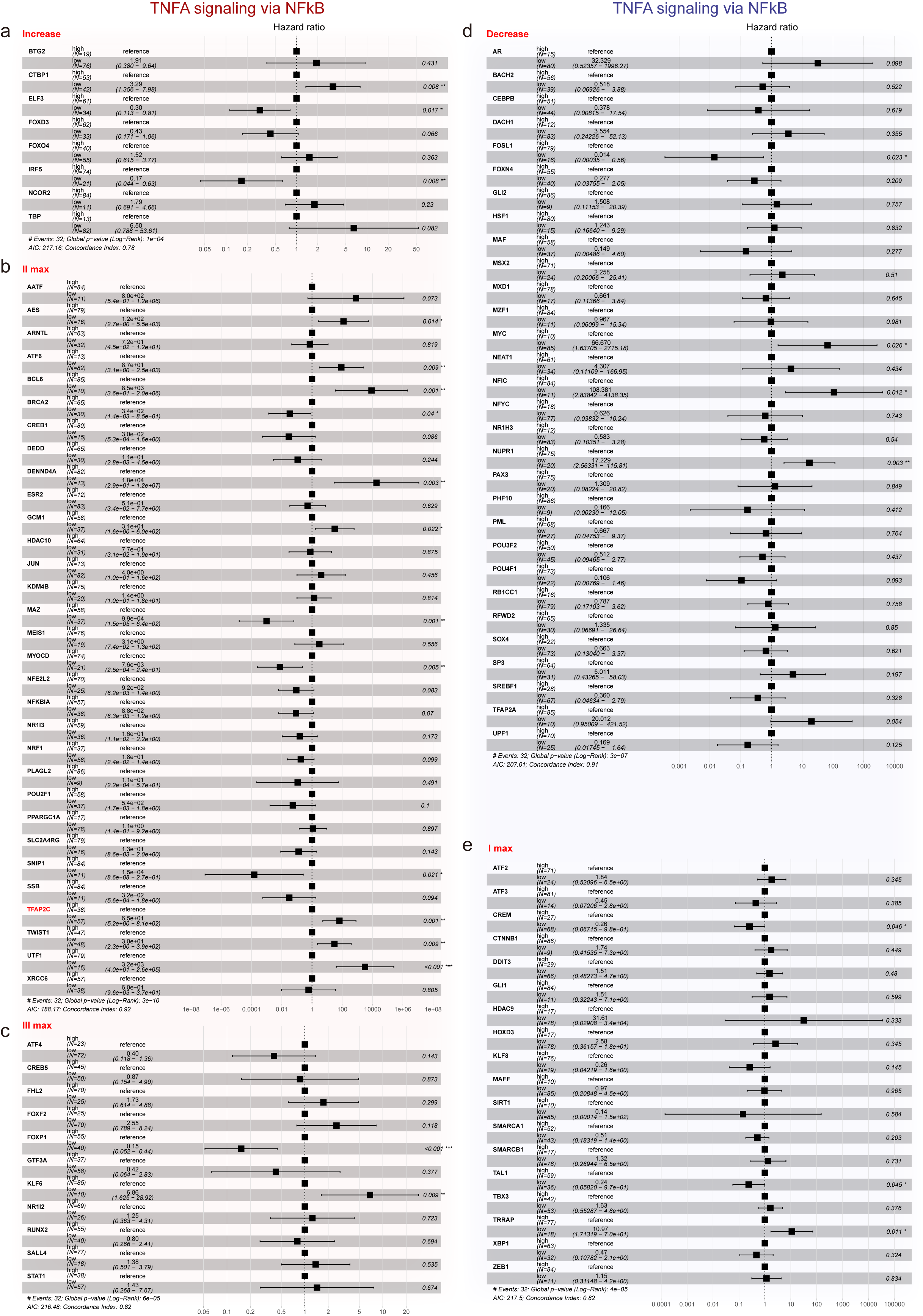

Supplement: Supplementary file 1 [file ijms-27-04433-s001.zip › Fig. S6.tif]

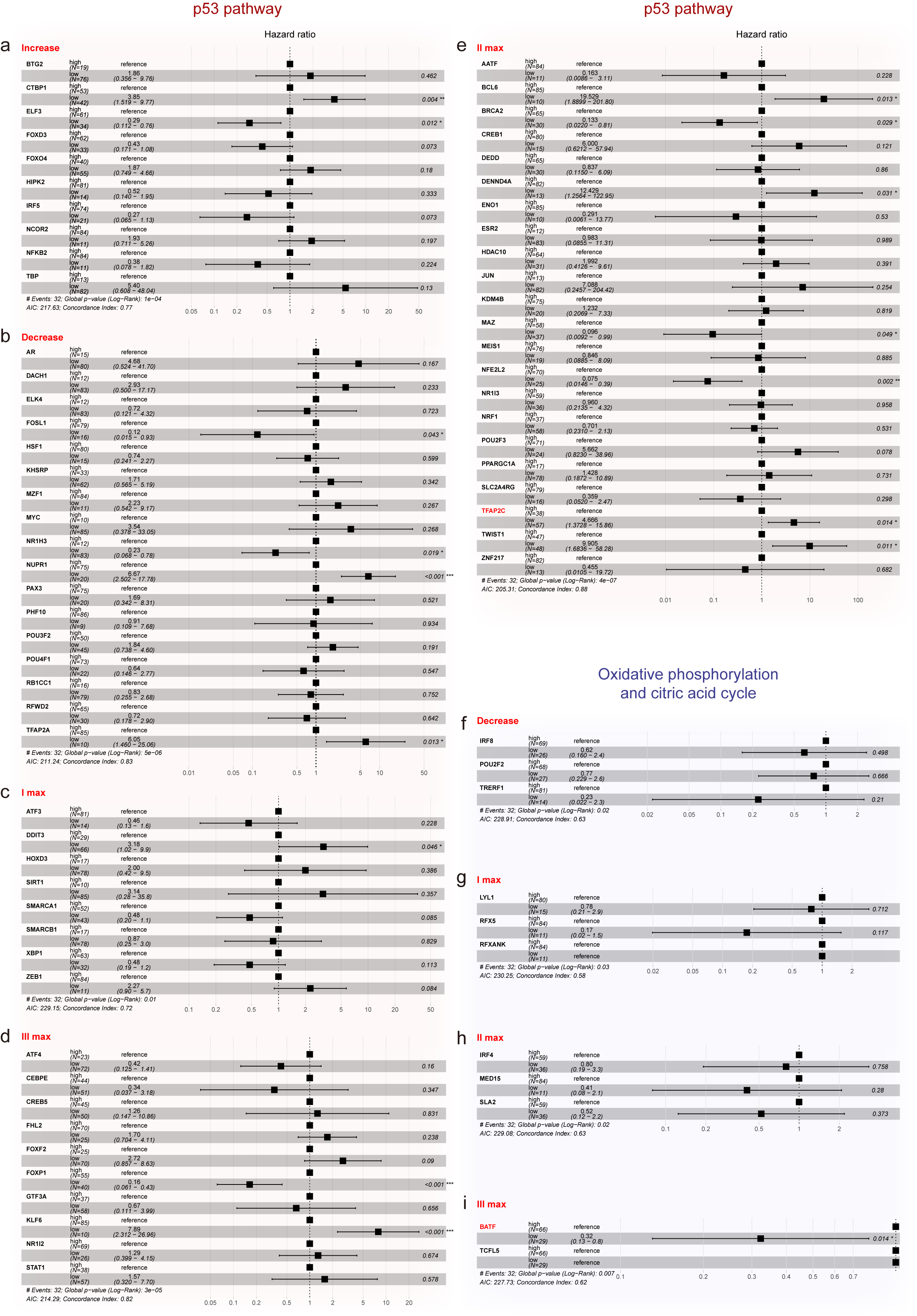

Supplement: Supplementary file 1 [file ijms-27-04433-s001.zip › Fig. S7.tif]

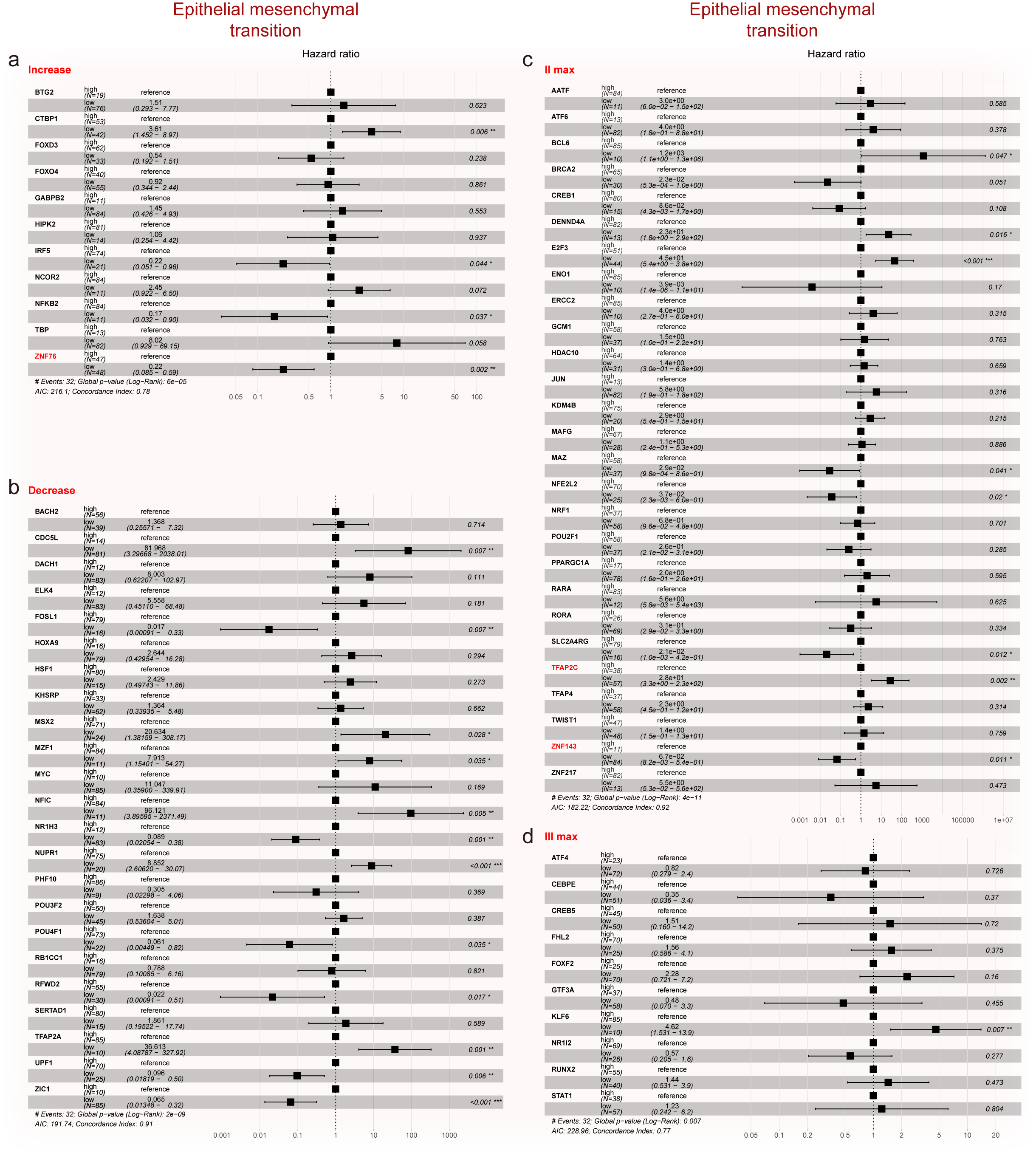

Supplement: Supplementary file 1 [file ijms-27-04433-s001.zip › Fig. S8.tif]

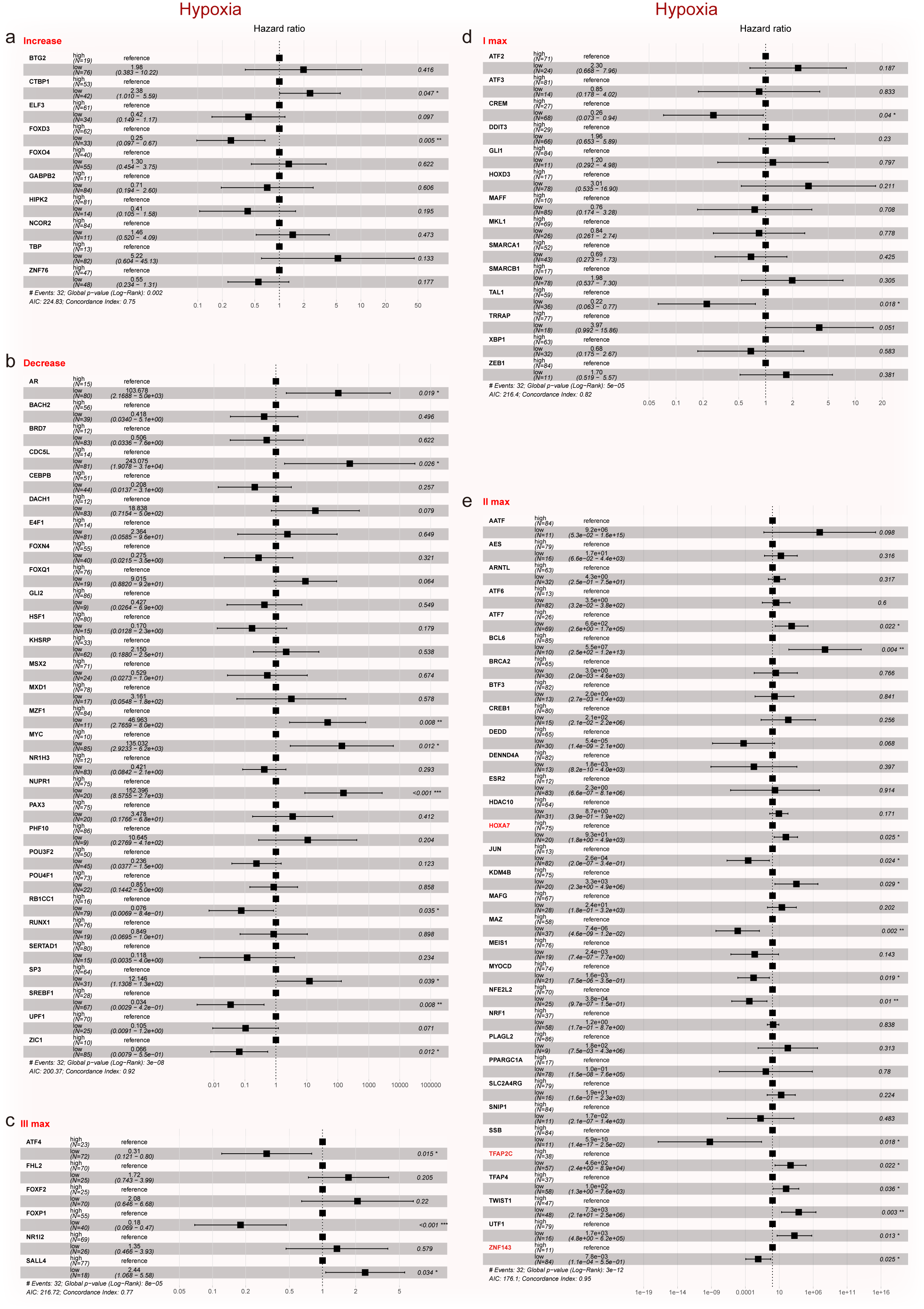

Supplement: Supplementary file 1 [file ijms-27-04433-s001.zip › Fig. S9.tif]

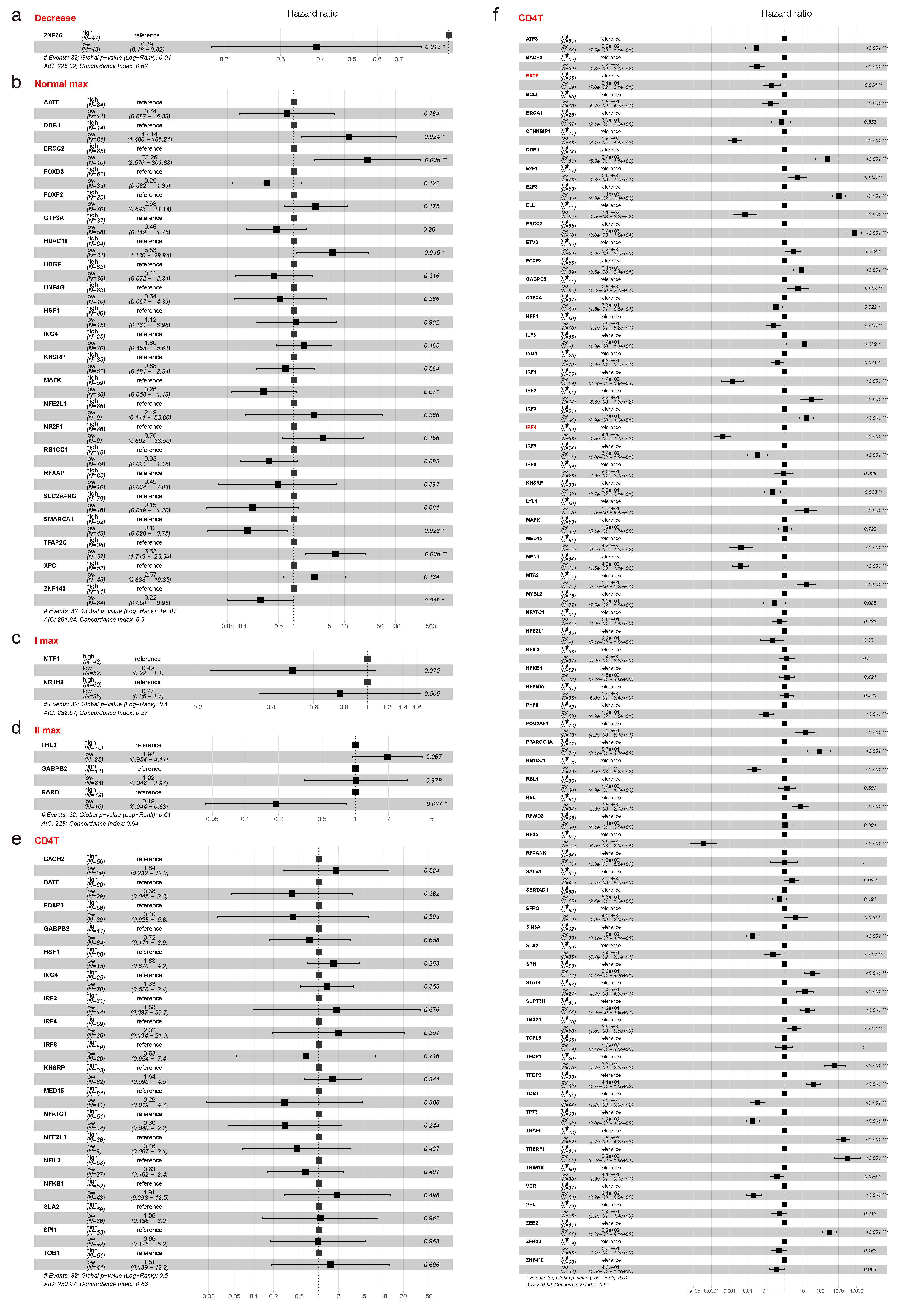

Supplement: Supplementary file 1 [file ijms-27-04433-s001.zip › Fig. S10.tif]

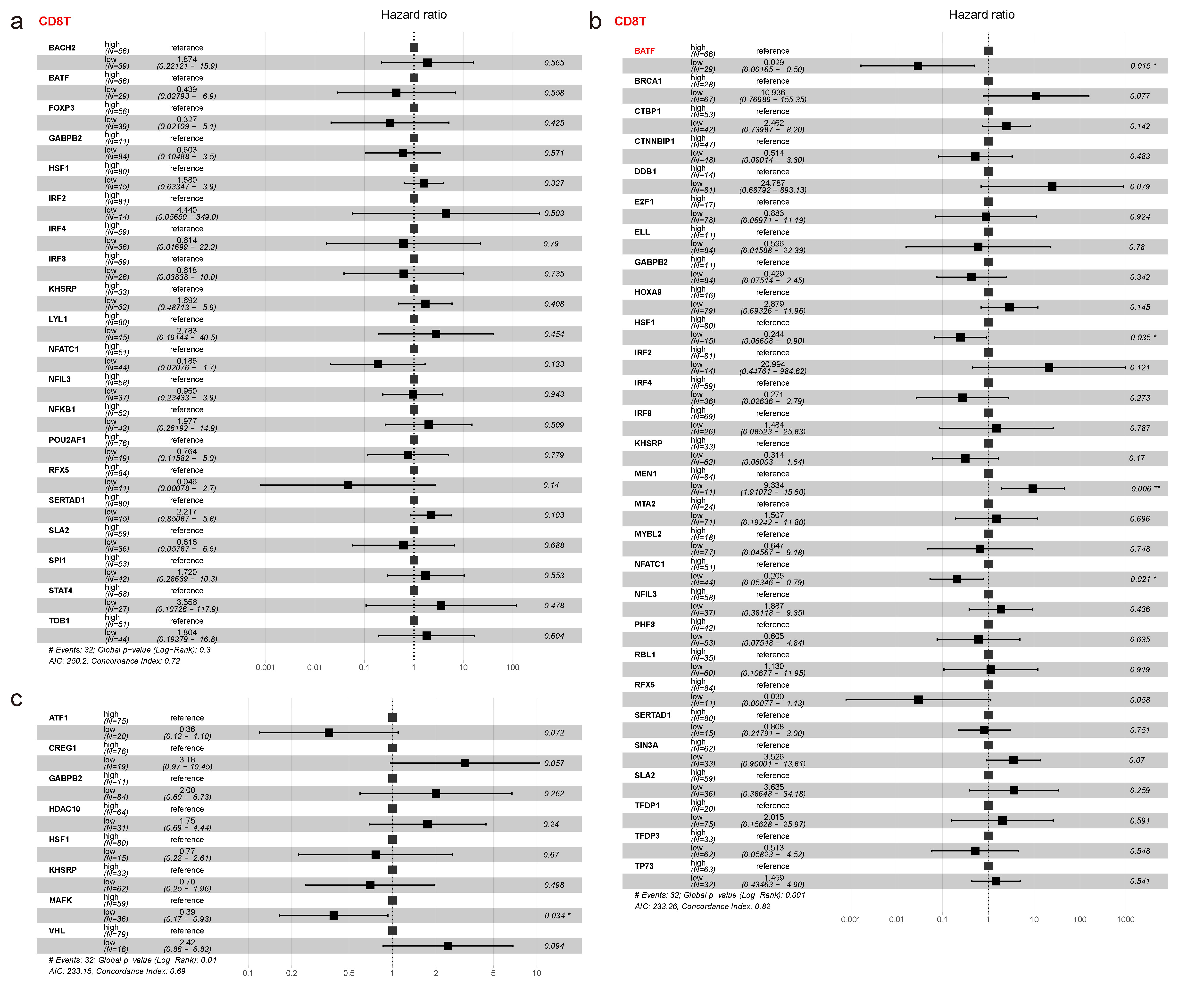

Supplement: Supplementary file 1 [file ijms-27-04433-s001.zip › Fig. S11.tif]
